# Advances in gene therapy for Lafora disease: Intravenous recombinant adeno‐associated virus‐mediated delivery of *EPM2A* and *EPM2B* genes

**DOI:** 10.1002/ctm2.70514

**Published:** 2025-10-30

**Authors:** Luis Zafra‐Puerta, Nerea Iglesias‐Cabeza, Miriam Sciaccaluga, Laura Bellingacci, Jacopo Canonichesi, Gema Sánchez‐Martín, Cinzia Costa, Marina P. Sánchez, José M. Serratosa

**Affiliations:** ^1^ Laboratory of Neurology Instituto de Investigación Sanitaria‐Fundación Jiménez Díaz Universidad Autónoma de Madrid (IIS‐FJD, UAM) Madrid Spain; ^2^ PhD Program in Neuroscience Universidad Autónoma de Madrid‐Cajal Institute Madrid Spain; ^3^ Fondazione Malattie Rare Mauro Baschirotto BIRD Onlus Longare Vicenza Italy; ^4^ Department of Life Sciences, Health, and Health Professions Link University Rome Italy; ^5^ Section of Physiology Department of Medicine and Surgery University of Perugia Perugia Italy; ^6^ Section of Neurophysiopathology S.M. Della Misericordia Hospital Laboratory of Experimental Neurology Department of Medicine and Surgery University of Perugia Perugia Italy

**Keywords:** Early treatment, *EPM2A*, *EPM2B*, gene therapy, intravenous administration, Lafora disease, laforin, malin, recombinant adeno‐associated virus

## Abstract

**Background:**

Lafora disease is a rare and fatal form of progressive myoclonus epilepsy that typically manifests in late childhood, presenting with seizures and progressive neurological decline. It is caused by mutations in *EPM2A* or *EPM2B* genes, encoding laforin and malin, which form a complex that regulates glycogen metabolism and mitigates cellular stress. Loss of function in either gene leads to the accumulation of Lafora bodies, insoluble polyglucosan aggregates that contribute to neurodegeneration.

**Methods:**

We previously demonstrated the efficacy of gene therapy using intracerebroventricular delivery of rAAV2/9 vectors expressing *EPM2A* or *EPM2B* in mouse models of Lafora disease. Building on these findings, we investigated the therapeutic and translational potential of a less invasive approach using intravenous delivery of rAAV2/9P31 vectors, which efficiently cross the blood–brain barrier. Gene delivery was performed at presymptomatic stages in *Epm2a* ^−/−^ and *Epm2b* ^−/−^ mice.

**Results:**

Intravenous gene therapy with rAAV2/9P31 vectors carrying *EPM2A* or *EPM2B* reversed neuropathological features of the disease, restored neuronal excitability and synaptic plasticity, and effectively prevented Lafora body formation. The therapeutic outcomes were comparable or superior to those achieved with intracerebroventricular administration. Long‐term evaluation revealed no evidence of hepatotoxicity or immunogenicity.

**Conclusion:**

Our results support intravenous rAAV2/9P31–mediated gene therapy as a promising, less invasive, and safe treatment strategy for Lafora disease, with strong potential for clinical translation.

## Graphical abstract

Intravenous gene therapy with rAAV2/9P31 vectors delivering *EPM2A* or *EPM2B* reversed Lafora disease pathology and restored neuronal function in two mouse models. The approach showed comparable or superior efficacy to intracerebroventricular delivery, with no long‐term toxicity, supporting its translational potential.

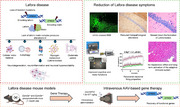

## 1 INTRODUCTION

Lafora disease (OMIM #254780; ORPHA: 501) is a rare and fatal neurodegenerative disorder that typically manifests in late childhood with seizures – mostly myoclonic and generalised tonic–clonic (GTC) seizures – motor dysfunction, and severe cognitive decline. Progressive neurological deterioration leads to dementia and death within 5 to 15 years of onset.^1^, ^2^, ^3^, ^4^ There is no specific therapy available, leaving patients dependent on antiseizure medications for temporary seizure management.

The disease is caused by loss‐of‐function mutations with an autosomal recessive inheritance pattern in *EPM2A* (OMIM 607566), which codes for laforin,^5^, ^6^, ^7^, ^8^ or *EPM2B* (OMIM 608072), encoding malin.^9^, ^10^, ^11^ Laforin, a dual‐specificity phosphatase, has glucan phosphatase activity, serves as an adaptor for glycogen‐metabolising enzymes, and regulates endoplasmic reticulum (ER) stress and protein clearance.^12^ Malin is an E3‐ubiquitin ligase that interacts with laforin to form a functional complex controlling proteins involved in glycogen metabolism, protein folding, autophagy, mitochondrial function, and oxidative stress.^11^, ^12^, ^13^, ^14^, ^15^, ^16^, ^17^, ^18^, ^19^, ^20^, ^21^, ^22^ Interaction of malin with target enzymes requires laforin.^14^, ^15^ Mutations affecting either protein trigger the accumulation of Lafora bodies (LBs), which consist of polyglucosans – abnormal, insoluble, poorly branched, and hyperphosphorylated glycogen – along with associated proteins.^23^, ^24^, ^25^, ^26^, ^27^ Beyond glycogen defects, the pathophysiology encompasses oxidative stress, protein misfolding, impaired autophagy, proteasomal dysfunction,^17^, ^18^, ^19^, ^20^, ^28^, ^29^, ^30^, ^31^, ^32^ and defective glutamate uptake,^33^, ^34^, ^35^ culminating in neuronal hyperexcitability, neurodegeneration and neuroinflammation.^33^, ^35^, ^36^, ^37^, ^38^, ^39^, ^40^, ^41^, ^42^, ^43^, ^44^

*Epm2a^−/−^* ^45^ and *Epm2b^−/−^* ^29^ knockout mice replicate the major neurological abnormalities observed in patients, albeit with milder phenotypes.^29^, ^45^, ^46^ In these models, various therapeutic strategies have been tested, including neuroprotective agents such as 4‐phenylbutyric acid (4‐PBA) and metformin, the latter currently used in patients to delay symptom onset and improve daily function,^47^, ^48^, ^49^ as well as sodium selenate,^50^ Myozyme^®^,^51^ VAL‐0417,^52^, ^53^ rAAV vectors carrying CRISPR/Cas9 or miRNA against glycogen synthase 1 gene *(Gys1)*,^52^, ^53^ antisense oligonucleotides targeting *Gys1* mRNA,^54^, ^55^ and modulators of neuroinflammation.^56^, ^57^

Our group has previously used the rAAV2/9‐CAG‐h*EPM2A* ^58^ and rAAV2/9‐CAG‐h*EPM2B* (under review) vectors administered intracerebroventricularly (ICV) in 3‐month‐old *Epm2a^−/−^* and *Epm2b^−/−^* mice. Although this route of administration bypasses the blood–brain barrier (BBB) and reaches central nervous system (CNS), distribution and transduction are often limited. To improve CNS targeting, AAV capsid variants have been engineered to enhance their ability to cross the BBB.^59^, ^60^, ^61^, ^62^ One such variant, 9P31, exhibits higher CNS transduction efficiency and improved BBB crossing.^59^, ^63^

In this study, we hypothesise that early intravenous (IV) delivery of the rAAV2/9P31‐CAG‐h*EPM2A* (rAAVP31‐h*EPM2A*) and rAAV2/9P31‐CAG‐h*EPM2B* (rAAVP31‐h*EPM2B*) vectors, encoding the respective human genes, will prevent or mitigate neurological and histopathological abnormalities, decrease epileptic activity, and reduce LB formation in *Epm2a^−/−^* and *Epm2b^−/−^* mice. We also assessed safety, including potential hepatic toxicity and immunogenicity, to determine feasibility for clinical translation.

## 2 MATERIALS AND METHODS

### 2.1 Experimental animals

The *Epm2a^−/−^* and *Epm2b^−/−^* mouse models of Lafora disease were generated as previously described.^29^, ^45^ Given that no gender‐specific phenotype variances have been observed in mice or patients with Lafora disease,^64^, ^65^ our analysis incorporated data from both male and female mice. The mouse colonies were bred at the Animal Facility Service of the Instituto de Investigación Sanitaria‐Fundación Jiménez Díaz and were housed individually in isolated cages. They were maintained under a 12:12 light/dark cycle at a constant temperature of 23°C, with ad libitum access to standard rodent chow and water. Experimental procedures were conducted with the utmost consideration for animal welfare, employing the minimum number of animals necessary while minimising potential distress. The protocols adhere to the guidelines outlined in the ‘Principles of Laboratory Animal Care’ (NIH publication No. 86–23, revised 1985), as well as the European Communities Council Directive (2010/63/EU). They received approval from the Ethical Review Board of the Instituto de Investigación Sanitaria‐Fundación Jiménez Díaz, and the Animal Care and Use Committee at the University of Perugia (authorisation no. 2B818.N.9JX). The ARRIVE guidelines were strictly followed.^66^

### 2.2 Generation of rAAV9P31‐h*EPM2A*, rAAV9P31‐h*EPM2B*, rAAV9P31‐*GFP*, and rAAV9P31‐Null vectors

The rAAVP31‐h*EPM2A* vector, which contains the cDNA for *EPM2A* transcript variant 1 (NM_005670.4), the rAAVP31‐h*EPM2B* vector, carrying the cDNA of the *EPM2B* gene (NM_198586.3), the rAAV2/9P31‐CAG‐*GFP* (rAAVP31‐*GFP*) vector, containing the green fluorescent protein (*GFP*) gene, and the rAAV2/9P31‐CAG‐Null (rAAVP31‐Null) vector, harbouring non‐coding DNA sequences, were produced by the Unitat de Producció de Vectors (UPV; www.viralvector.eu) using the triple transfection method. In addition, HA‐tagged versions of the therapeutic constructs, rAAV2/9P31‐CAG‐HA‐h*EPM2A* (rAAVP31‐HA‐h*EPM2A)* and rAAV2/9P31‐CAG‐HA‐h*EPM2B* (rAAVP31‐HA‐h*EPM2B)*, were similarly designed and produced to assess the distribution of the therapeutic proteins. This process utilised three plasmids: (1) one containing inverted terminal repeat (ITR) sequences, (2) one encoding the AAV capsid proteins (VP1, VP2, and VP3) along with replication genes, and (3) an adenoviral helper plasmid. Following production, the vectors were purified by iodixanol‐based ultracentrifugation to remove empty capsids.^67^

### 2.3 Intravenous injections

At 1 month of age, wild‐type (WT), *Epm2a^−/−^* and *Epm2b^−/−^* mice were anaesthetised with 4% isoflurane and received a single IV injection into the retro‐orbital venous sinus with rAAVP31‐h*EPM2A*, rAAVP31‐HA‐h*EPM2A*, rAAVP31‐h*EPM2B*, rAAVP31‐HA‐h*EPM2B*, rAAVP31‐*GFP* or rAAVP31‐Null vectors. The total volume injected was 50 µL, and the dose was 1.9 × 10^12^ vg/kg. Results were analysed 5 months post‐injection. Between 25 and 35 mice in each group and condition were used.

### 2.4 RNA extraction and quantitative reverse transcription‐polymerase chain reaction

Brain samples were homogenised on ice using TRIzol™ Reagent (ThermoFisher Scientific, Massachusetts, USA). The extracted RNA pellets underwent washing, drying, resuspension, and treatment with DNase Enzyme (ThermoFisher Scientific, Massachusetts, USA) before quantification using a NanoDrop ND‐1000 spectrophotometer (ThermoFisher, Massachusetts, USA). The reverse transcription‐polymerase chain reaction (RT‐PCR) experiments were conducted using the High‐Capacity cDNA Reverse Transcription Kit with RNase inhibitor (ThermoFisher Scientific, Massachusetts, USA), employing 1 µg of RNA per reaction. The RT‐PCR conditions were 25°C for 10 min, 37°C for 120 min, and 85°C for 5 min. For quantitative RT‐PCR (RT‐qPCR), cDNA from the h*EPM2A* transcript variant 1 and the h*EPM2B* transcript served as templates. The reaction was performed using TaqMan™ Fast Advanced Master Mix (ThermoFisher Scientific, MA, USA), along with *Epm2a*, *EPM2A, Epm2b, EPM2B*, and glyceraldehyde 3‐fosfato dehydrogenase (*Gapdh*) probes (ThermoFisher Scientific, MA, USA). The RT‐qPCR conditions were 50°C for 2 min, 95°C for 2 min, followed by 40 cycles of 95°C for 1 s and 60°C for 20 s. Analysis was carried out using the 2^−ΔΔCT^ method.

### 2.5 Motor coordination

Motor coordination and balance were assessed using the rotarod test (Harvard Apparatus, Holliston, MA, USA) following previously described methods.^40^, ^58^

### 2.6 Object recognition task

The object recognition task (ORT) was used to assess episodic memory retention according to previously published protocol.^40^, ^58^

### 2.7 Sensitivity to pentylenetetrazole

To investigate neuronal hyperexcitability, petylenetetrazole (PTZ) was administered via intraperitoneal injection at 30 mg/kg. Each animal was observed for 45 min by two researchers.

### 2.8 Electrophysiology

#### 2.8.1 Brain slicing

Mice were sacrificed by cervical dislocation. The brain was collected and immersed in ice‐cold artificial cerebrospinal fluid (ACSF) containing (in mM): 126 NaCl, 2.5 KCl, 1.2 MgCl_2_, 1.2 NaH_2_PO_4_, 2.4 CaCl_2_, 10 glucose, and 25 NaHCO_3_, continuously bubbled with 95% O_2_ and 5% CO_2_, pH 7.4. Hippocampal transverse slices (400 µm thick for extracellular recordings) and cortico‐striatal coronal slices (220 µm thick for patch‐clamp recording) were obtained using a vibratome (LEICA, VT 1200S). Hippocampal brain slices were maintained in iced ACSF solution during the cutting process, while striatal brain slices were maintained in ACSF solution, bubbled with a 95% O_2_–5% CO_2_ gas mixture, at room temperature. Once cut, the slices were transferred to a recovery chamber with oxygenated ACSF at 30°C for 30 min and then kept at room temperature^68^ for 1–2 more hours before experimental recordings. Each slice was then transferred to the recording chamber and submerged in ACSF at a constant rate of 2.5–3 mL/min at 29°C for the hippocampus and at RT for the striatum.

#### 2.8.2 Extracellular recording

The stimulating electrode was inserted into the perforant path fibres, and the recording electrode, made of borosilicate glass capillaries filled with 2 M NaCl (resistance 10–15 MΩ), was placed in the dentate gyrus (DG) close to the granular layer. Stimuli of  .1 Hz, 10 ms duration, and 20–30 V amplitude evoked field excitatory post‐synaptic potentials (fEPSPs), which in the DG included a PS that reached 50% of maximum amplitude. The PS amplitude was defined as the average of the amplitude from the early positive peak to the negative peak, and from the negative peak to the late positive peak. An Axoclamp 2B amplifier (Molecular Devices, USA) was used for extracellular recordings. Traces were filtered at 3 KHz, digitised at 10KHz, and stored on a PC.

To induce long‐term potentiation (LTP) in the DG, a high‐frequency stimulation (HFS) protocol was delivered^69^ after acquiring a stable baseline for 10 min.

#### 2.8.3 Evaluation of epileptic‐like activity in DG

Epileptic‐like activity in DG was induced by perfusing slices with an Mg^2+^‐free external solution to remove the magnesium block from N‐Methyl‐D‐Aspartate (NMDA) glutamate receptors, in presence of bicuculline to antagonise gamma‐aminobutyric acid A (GABA_A_) receptors.^70^ The epileptic‐like activity was measured as the mean number of the PS and as PS amplitude (%). Specifically, for each experiment, the individual sweeps were averaged, yielding an average trace every 2 min. For each average trace obtained, the sum of the amplitudes of the PSs (considering from the second peak onwards) was normalised to the amplitude of the first peak, and the value was reported as a percentage. Thus, a time course of the amplitude of the PSs in percentage was obtained.

#### 2.8.4 Whole‐cell patch‐clamp recording

Whole‐cell patch‐clamp recordings were performed from medium spiny neurons (MSNs) visualised using infrared differential interference contrast microscopy (Olympus) in the dorsolateral striatum. Whole‐cell patch‐clamp recordings (access resistance: 15–30 MΩ; holding potential: −80 mV) were performed using a Multiclamp 700B amplifier (Molecular Devices) with borosilicate glass pipettes (4–7 MΩ) filled with an internal solution containing 145 mM K+‐gluconate,  .1 mM CaCl_2_, 2 mM MgCl_2_,  .1 mM EGTA, 10 mM HEPES,  .3 mM Na‐GTP and 2 mM Mg‐ATP, adjusted to pH 7.3 with KOH. Access resistance was monitored online throughout each experiment, and recordings were discarded if either access resistance or holding current increased by more than 25% during the experiment. No liquid junction potential correction was applied. Membrane capacitance and resistance were measured online using the membrane seal test function of pClamp 10.7 (–5 mV step, 10 ms).

MSNs were identified by their hyperpolarised resting membrane potential (RMP, ∼ −80 mV), absence of spontaneous action potential discharge at rest, and presence of tonic firing activity during current‐induced membrane depolarisation. When recording spontaneous excitatory postsynaptic currents (sEPSCs), picrotoxin (50 µM) was added to the ACSF to block GABA_A_ currents. Data were acquired with pClamp 10.7 (Molecular Devices), with currents filtered at  .1 kHz and digitised at 200 µs using Clampex 10.7. The data were analysed offline using the automatic detection and subsequently checked manually for accuracy. For MSNs synaptic plasticity recordings, a bipolar electrode, connected to a stimulation unit was placed in the white matter between the cortex and the striatum to stimulate glutamatergic fibres (.1 Hz) and evoked eEPSCs, while the recording electrode was placed in the dorsolateral striatum. After recording eEPSCs of stable amplitudes for at least 10 min, a HFS protocol, consisting of three trains of 3 s (20 s intervals), was delivered at 100 Hz to induce LTP. External Mg^2+^ ions were omitted from the solution to maximise the contribution of NMDA receptors during LTP experiments. In all patch‐clamp experiments, 50 µM picrotoxin was added to the Krebs solution to block GABA_A_ receptors.

### 2.9 Periodic‐acid Schiff‐diastase staining, immunohistochemistry and haematoxylin‐eosin staining

Following previous protocols,^40^, ^58^ mice were anaesthetised and transcardially perfused with 4% phosphate‐buffered paraformaldehyde. Their tissues were treated with porcine pancreas α‐amylase (5 mg/mL in dH2O) (Merck, Darmstadt, Germany) and processed using the periodic‐acid Shiff (PAS) Kit (Merck, Darmstadt, Germany) or directly stained with haematoxylin and eosin (H&E) (PHC Holdings, Epredia Inc., Chiyoda, Tokyo, Japan). For PAS‐diastase (PAS‐D) staining, sections were counterstained with Gill No. 3 haematoxylin (Merck, Darmstadt, Germany).

For immunohistochemistry (IHC), the rehydrated sections were incubated with different primary antibodies, included glial fibrillary acidic protein (GFAP; 1:1000 dilution; Millipore, Temecula, CA, USA; Cat. #MAB360), neuronal nuclei (NeuN; 1:100 dilution; Millipore, Temecula, CA, USA; Cat. # MAB377) and HA‐Tag (C29F4) (1:1600; Cell Signaling Technology, Danvers, MA, USA; Cat. #3724S). Sections were then incubated with biotinylated anti‐mouse and anti‐rabbit IgG and stained using the Vectastain ABC kit (Vector Laboratories, Burlingame, CA, USA). Immunoreactivity was visualised with diaminobenzidine (Dako Cytomation, CA, USA) and H_2_O_2_, followed by counterstaining with Carazzi haematoxylin (Panreac Quimica, Barcelona, Spain). Samples from 4–6 mice per group were used, with two consecutive sections per animal analysed. Images were acquired using a Leica DMLB 2 microscope (Leica, Wetzlar, Germany) equipped with a Leica DFC320 FireWire digital microscope camera (Leica, Wetzlar, Germany). Quantification of LBs, GFAP‐, and NeuN‐positive cells was performed by two researchers using ImageJ software (NIH, Bethesda, MD, USA). The reported values represent the average of these quantifications. The examination of histological changes in the liver was focused on evaluating lobular inflammation, scoring it based on the Non‐Alcoholic Fatty Liver Disease (NAFLD) activity score criteria.^71^

### 2.10 Biochemical parameters

Glucose levels, liver biochemical parameters and immunoglobulin profile, including aspartate aminotransferase,^72^ alanine aminotransferase (ALT), alkaline phosphatase (ALP), immunoglobulin G (IgG) and immunoglobulin M (IgM), were assessed using a Roche Cobas autoanalyser.

### 2.11 Statistical analysis

Data are expressed as mean ± standard error of the mean (SEM) or as percentages. To assess differences between experimental groups, we used one‐way ANOVA, Fisher's exact test, the non‐parametric Kruskal–Wallis test, or the Mann–Whitney test, as indicated for each specific case. Statistical analyses were performed using GraphPad Prism 8.0 (San Diego, CA). Statistical significance thresholds were denoted as *p < .05, **p < .01, ***p < .001, and ****p < .0001.

Data analysis for electrophysiological recordings was performed offline using Clampfit 10.7 (Molecular Devices) and GraphPad Prism 9.0 (GraphPad Software, Inc., La Jolla, CA 92037, USA). Values given in the text and figures are expressed as means ± SEM, with ‘*n*’ representing the number of cells or field potentials recorded. Two‐way ANOVA or Student's *t*‐test was used for statistical analysis. The significance level was established at *p < .05.

## 3 RESULTS

### 3.1 Vectors transduction leads to expression of both transgenes within CNS cells and other organs

**FIGURE 1:**
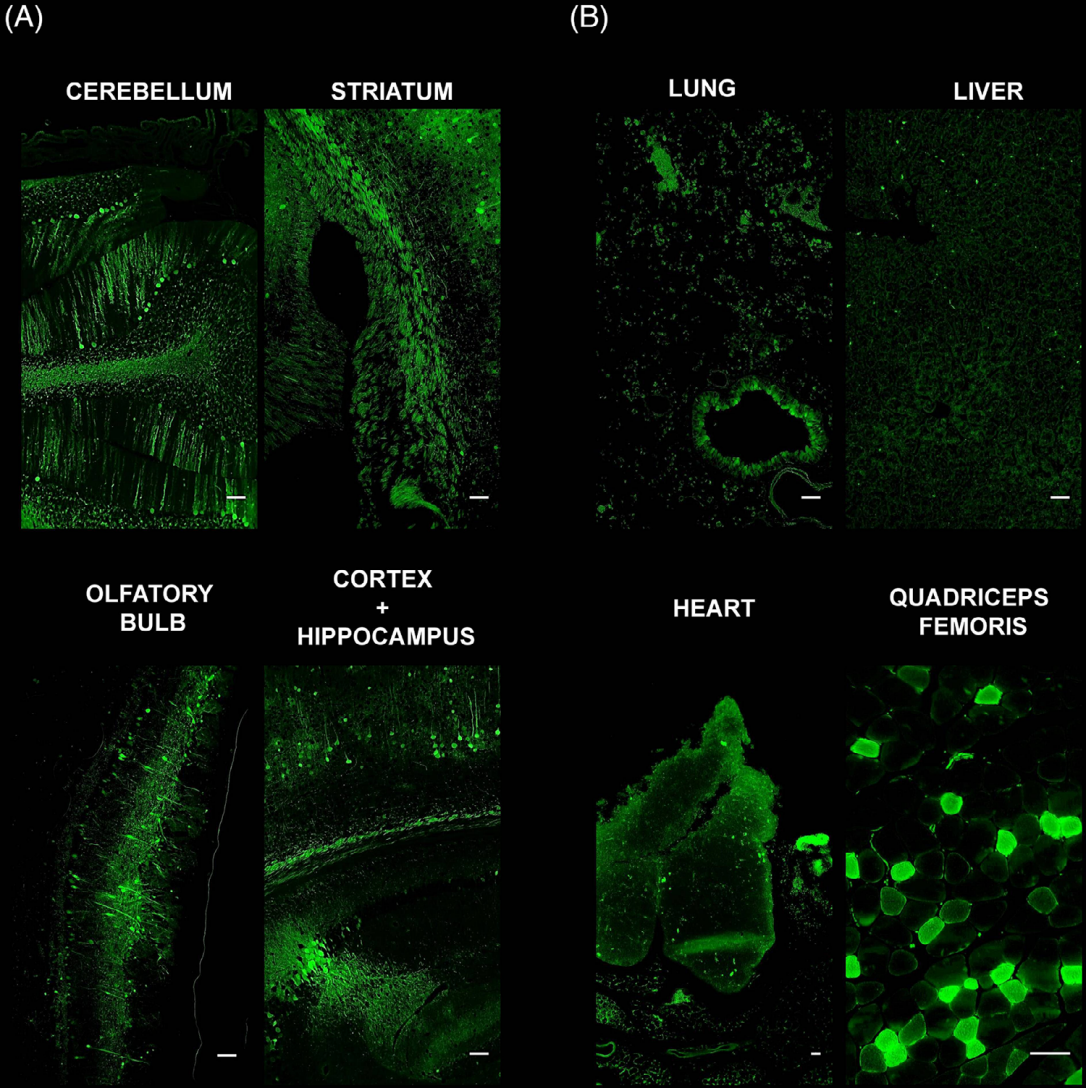
Expression of the rAAVP31‐*GFP* vector in the brain and other tissues of WT mice 5 months post‐IV injection. (A) Reporter GFP protein demonstrates transduction of CNS cells in the cerebellum, striatum, olfactory bulb, cortex and hippocampus. (B) Images of GFP protein expression illustrate the ability of the vector to transduce cells and express the GFP protein in diverse tissues, such as the lungs, liver, heart, and quadriceps femoris of WT mice. Scale bars = 50 µm.

**FIGURE 2:**
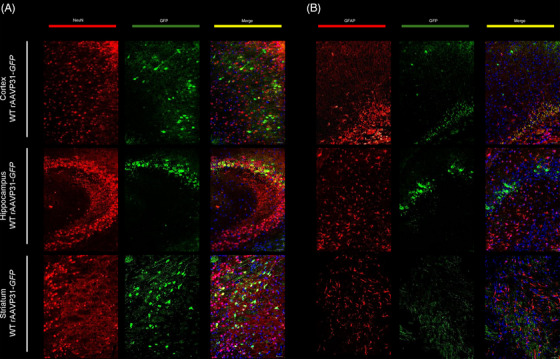
Cellular tropism of the rAAVP31‐GFP vector in the CNS of WT mice. (A, B) Double immunofluorescence analysis using (A) neuronal (NeuN, red) and (B) glial (GFAP, red) markers to characterise the cell types transduced by the vector. (A) GFP‐positive cells (green) predominantly colocalise with NeuN, indicating strong neuronal tropism, whereas minimal colocalisation with GFAP was observed, showing low glial transduction. Scale bars = 25 µm.

We used the rAAVP31‐*GFP* vector to confirm its ability to cross the BBB. Five months after a single IV injection in 1‐month‐old WT mice, we observed widespread biodistribution in the CNS, including the cerebellum, striatum, olfactory bulb, cortex, hippocampus (Figure 1A), and spinal cord (data not shown), as well as in other peripheral organs such as the lungs, liver, heart, and quadriceps femoris muscle (Figure 1B). To further characterise the cellular tropism of the vector within the CNS, we performed double immunofluorescence staining using neuronal (NeuN) and glial (GFAP) markers. This analysis revealed that the vast majority of GFP‐positive cells were neurons, indicating a predominant neuronal tropism of the rAAVP31‐*GFP* vector, with minimal transduction observed in glial cells (Figure 2).

**FIGURE 3:**
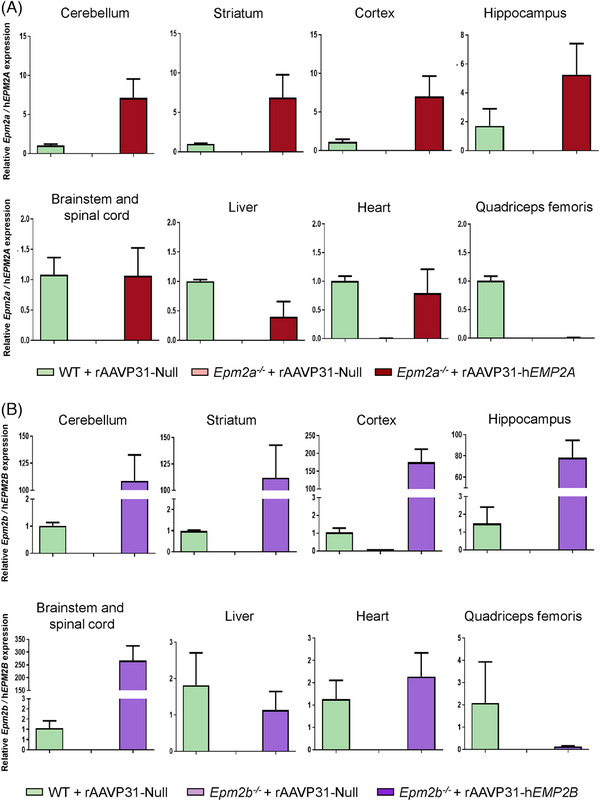
RT‐qPCR quantification of human *EPM2A* and *EPM2B* cDNAs in different areas of the CNS and in the heart, liver and quadriceps femoris, 5 months after a single IV injection of the rAAVP31‐h*EPM2A* or rAAVP31‐h*EPM2B* vectors in *Epm2a^−/−^* or *Epm2b^−/−^* mice. (A, B) RT‐qPCR quantification showing the levels of *EPM2A* (A) or *EPM2B* (B) cDNAs in different regions of the CNS, as well as in the heart, liver and quadriceps femoris samples collected from WT, *Epm2a^−/−^* (A) and *Epm2b^−/−^* (B) mice, 5 months after IV injection of rAAVP31‐h*EPM2A* (A), rAAVP31‐h*EPM2B* (B) or rAAVP31‐Null vectors. The gene expression levels were normalised to the values observed for the *Gadph* gene. Data are shown as mean ± SEM. *n* = 3–4 mice per group and experiment.

**FIGURE 4:**
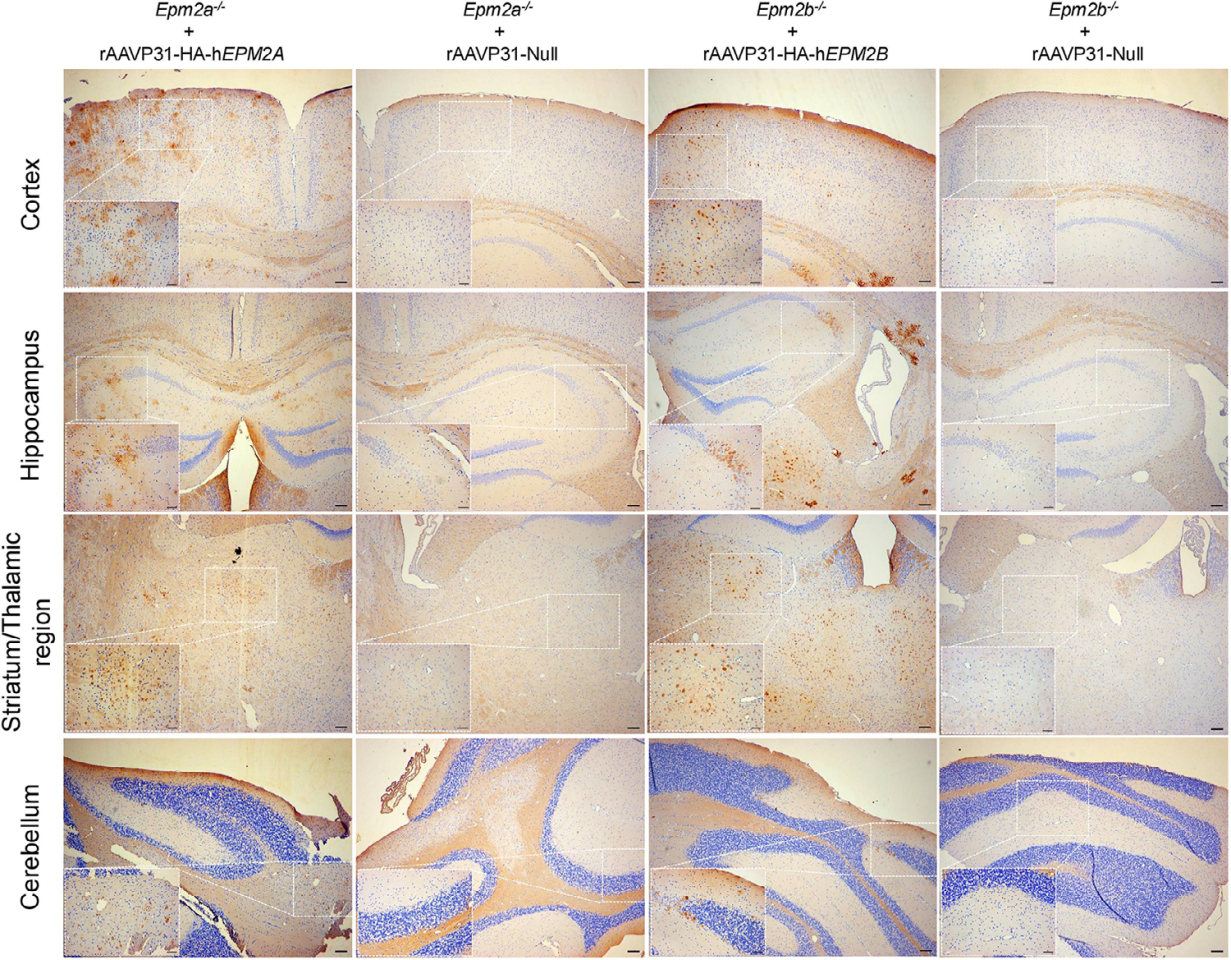
Distribution of human laforin and malin after a single IV injection of rAAVP31‐HA‐h*EPM2A* and rAAVP31‐HA‐h*EPM2B*. IHC with an anti‐HA antibody showing the distribution of the HA‐tagged human laforin and malin proteins across the cortex, hippocampus, striatum, thalamic regions and cerebellum. No expression was detected in *Epm2a^−/−^* and *Epm2b^−/−^* mice injected with the rAAVP31‐Null vector. Scale bars = 100 µm (overview images at 5×) and 50 µm (zoomed images at 20×).

Subsequently, transcription of the h*EPM2A* and h*EPM2B* transgenes was quantified via RT‐qPCR, 5 months after a single IV injection of the rAAVP31‐h*EPM2A* or rAAVP31‐*hEPM2B* vectors in *Epm2a^−/−^* and *Epm2b^−/−^* mice (Figure 3). The presence of h*EPM2A* (Figure 3A) and h*EPM2B* (Figure 3B) transcripts was confirmed throughout the CNS, including cerebellum, striatum, cortex and hippocampus, brainstem and spinal cord, as well as in other organs such as the liver and heart, but not in the quadriceps femoris. The expression levels of h*EPM2A* and h*EPM2B* transcripts were above the endogenous expression levels of the *Epm2a* and *Epm2b* genes in WT mice (Figure 3A and B), with a more pronounced difference observed in the case of *Epm2b^−/−^* mice (Figure 3B). Translation into protein was subsequently assessed using an anti‐HA antibody. A broad distribution of the therapeutic proteins was observed throughout the CNS, with similar detectable expression in the cortex, hippocampus, striatum, thalamus, and cerebellum (Figure 4).

### 3.2 Gene therapy improves motor coordination and memory deficits while decreasing sensitivity to PTZ

**FIGURE 5:**
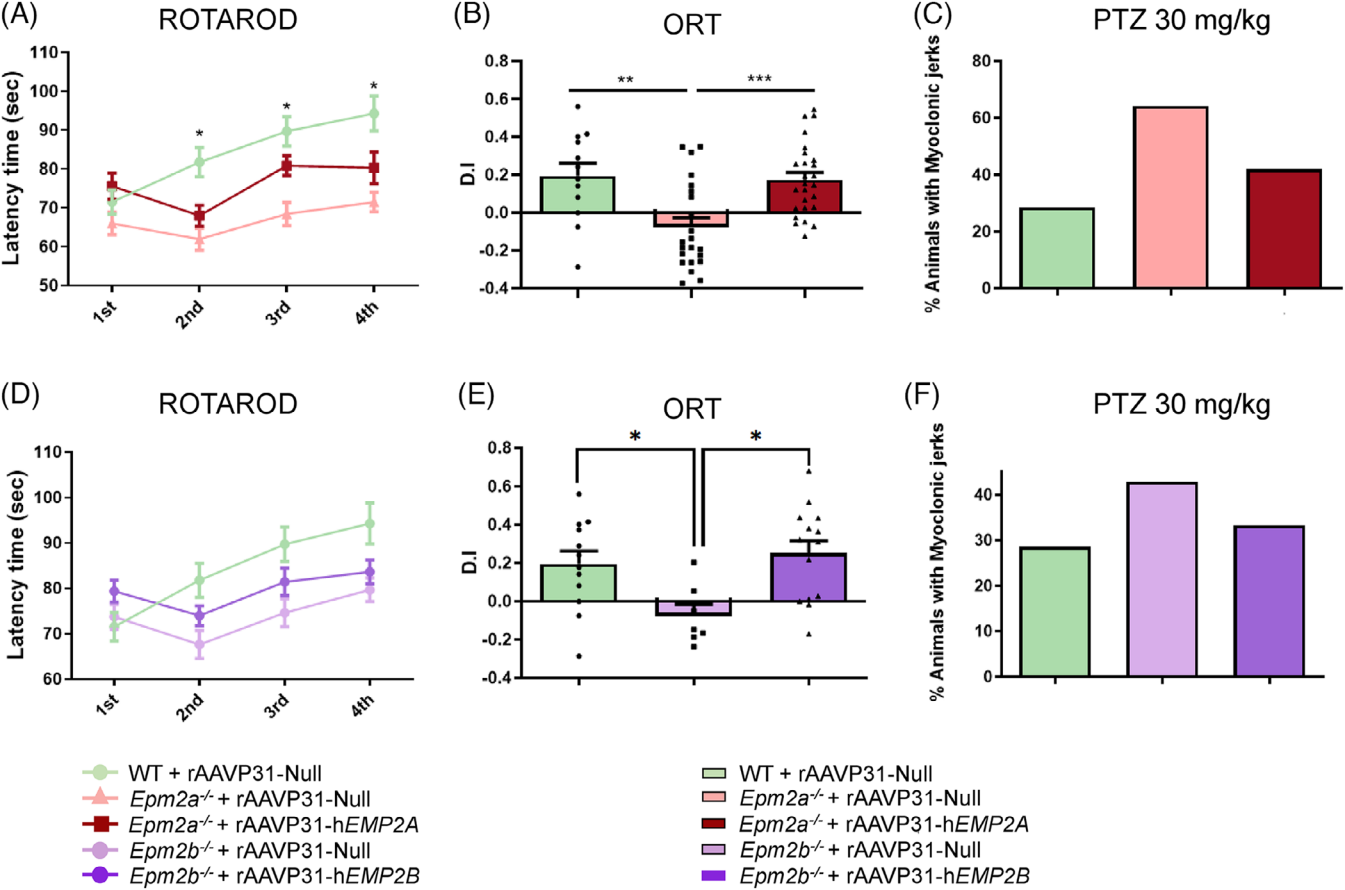
Behavioural studies and susceptibility to PTZ in WT, *Epm2a^−/−^* and *Epm2a^−/−^* mice, 5 months after IV injection of rAAVP31‐Null, rAAVP31‐h*EPM2A* or rAAVP31‐h*EPM2B* vectors. (A, D) Latency time for *Epm2a^−/−^* (A) and *Epm2b^−/−^* (D) mice to fall from the rotarod cylinder. (1st attempt: WT+rAAVP31‐Null vs. *Epm2a^−/−^* +rAAVP31‐Null *p*‐value = .7600; WT+rAAVP31‐Null vs. *Epm2a^−/−^* +rAAVP31‐h*EPM2A p*‐value = .8651; *Epm2a^−/−^* +rAAVP31‐Null vs. *Epm2a^−/−^* +rAAVP31‐h*EPM2A p*‐value = .45679; WT+rAAVP31‐Null vs. *Epm2b^−/−^* +rAAVP31‐Null *p*‐value = .9553; WT+rAAVP31‐Null vs. *Epm2b^−/−^* +rAAVP31‐h*EPM2B p*‐value = .4634; *Epm2b^−/−^* +rAAVP31‐Null vs. *Epm2b^−/−^* +rAAVP31‐h*EPM2B p*‐value = .7270/2nd attempt: WT+rAAVP31‐Null vs. *Epm2a^−/−^* +rAAVP31‐Null *p*‐value = .0316; WT+rAAVP31‐Null vs. *Epm2a^−/−^* +rAAVP31‐h*EPM2A p*‐value = .1850; *Epm2a^−/−^* +rAAVP31‐Null vs. *Epm2a^−/−^* +rAAVP31‐h*EPM2A p*‐value = .7224; WT+rAAVP31‐Null vs. *Epm2b^−/−^* +rAAVP31‐Null *p*‐value = .1864; WT+rAAVP31‐Null vs. *Epm2b^−/−^* +rAAVP31‐h*EPM2B p*‐value = .4966; *Epm2b^−/−^* +rAAVP31‐Null vs. *Epm2b^−/−^* +rAAVP31‐h*EPM2B p*‐value = .6895/3rd attempt: WT+rAAVP31‐Null vs. *Epm2a^−/−^* +rAAVP31‐Null *p*‐value = .0248; WT+rAAVP31‐Null vs. *Epm2a^−/−^* +rAAVP31‐h*EPM2A p*‐value = .5157; *Epm2a^−/−^* +rAAVP31‐Null vs. *Epm2a^−/−^* +rAAVP31‐h*EPM2A p*‐value = .2828; WT+rAAVP31‐Null vs. *Epm2b^−/−^* +rAAVP31‐Null *p*‐value = .2440; WT+rAAVP31‐Null vs. *Epm2b^−/−^* +rAAVP31‐h*EPM2B p*‐value = .5581; *Epm2b^−/−^* +rAAVP31‐Null vs. *Epm2b^−/−^* +rAAVP31‐h*EPM2B p*‐value = .7314/4th attempt: WT+rAAVP31‐Null vs. *Epm2a^−/−^* +rAAVP31‐Null *p*‐value = .0389; WT+rAAVP31‐Null vs. *Epm2a^−/−^* +rAAVP31‐h*EPM2A p*‐value = .3154; *Epm2a^−/−^* +rAAVP31‐Null vs. *Epm2a^−/−^* +rAAVP31‐h*EPM2A p*‐value = .5795; WT+rAAVP31‐Null vs. *Epm2b^−/−^* +rAAVP31‐Null *p*‐value = .2783; WT+rAAVP31‐Null vs. *Epm2b^−/−^* +rAAVP31‐h*EPM2B p*‐value = .3955; *Epm2b^−/−^* +rAAVP31‐Null vs. *Epm2b^−/−^* +rAAVP31‐h*EPM2B p*‐value = .9037). Symbols indicate: *WT+rAAVP31‐Null vs. *Epm2a^−/−^* +rAAVP31‐Null. (B, E) Memory assessment based on discrimination index evaluation in the ORT of *Epm2a^−/−^* (B), and of *Epm2b^−/−^* (E) mice, 5 months post‐IV injections of rAAVP31‐h*EPM2A*, rAAVP31‐h*EPM2B* or rAAVP31‐Null vectors. (WT+rAAVP31‐Null vs. *Epm2a^−/−^* +rAAVP31‐Null *p*‐value = .0027; WT+rAAVP31‐Null vs. *Epm2a^−/−^* +rAAVP31‐h*EPM2A p*‐value = .9652; *Epm2a^−/−^* +rAAVP31‐Null vs. *Epm2a^−/−^* +rAAVP31‐h*EPM2A p*‐value = .0005; WT+rAAVP31‐Null vs. *Epm2b^−/−^* +rAAVP31‐Null *p*‐value = .0479; WT+rAAVP31‐Null vs. *Epm2b^−/−^* +rAAVP31‐h*EPM2B p*‐value = .7956; *Epm2b^−/−^* +rAAVP31‐Null vs. *Epm2b^−/−^* +rAAVP31‐h*EPM2B p*‐value = .0112). Data are shown as mean ± SEM. A one‐way ANOVA test with Tukey's multiple comparisons was performed between the experimental groups. *p < .05, **p < .01, ***p < .001. (C, F) Percentage of animals exhibiting myoclonic jerks following intraperitoneal injection of 30 mg/kg PTZ in WT and *Epm2a^−/−^* (C) and *Epm2b^−/−^* (F) mice, 5 months after rAAV treatments. (WT+rAAVP31‐Null vs. *Epm2a^−/−^* +rAAVP31‐Null *p*‐value = .1283; WT+rAAVP31‐Null vs. *Epm2a^−/−^* +rAAVP31‐h*EPM2A p*‐value = .4861; *Epm2a^−/−^* +rAAVP31‐Null vs. *Epm2a^−/−^* +rAAVP31‐h*EPM2A p*‐value = .2960; WT+rAAVP31‐Null vs. *Epm2b^−/−^* +rAAVP31‐Null *p*‐value = .6946; WT+rAAVP31‐Null vs. *Epm2b^−/−^* +rAAVP31‐h*EPM2B p*‐value = > .9999; *Epm2b^−/−^* +rAAVP31‐Null vs. *Epm2b^−/−^* +rAAVP31‐h*EPM2B p*‐value = .7104). Data are shown as percentages. Statistical analysis was conducted using a Fisher's exact test. *n* = 12–20 mice per group.

We analysed the effects of the rAAVP31‐h*EPM2A* (Figure 5A–C) and rAAVP31‐h*EPM2B* (Figure 5D–F) vectors on motor coordination, episodic memory, and sensitivity to PTZ 5 months after IV injection in *Epm2a^−/−^* and *Epm2b^−/−^* mice.

Rotarod test revealed that *Epm2a^−/−^* mice injected with rAAVP31‐Null exhibited a significantly reduced latency to fall from the accelerating rod compared to WT controls, indicating impaired motor coordination. Treatment with rAAVP31‐h*EPM2A* in *Epm2a^−/−^* mice increased the latency to fall, eliminating the significant difference with WT animals (Figure 5A). *Epm2b^−/−^* mice also displayed reduced latency to fall from the rod compared to WT controls, although this difference did not reach statistical significance. Treatment with rAAVP31‐h*EPM2B* showed a trend towards improvement (Figure 5D). Episodic or short‐term memory performance was significantly enhanced in both *Epm2a^−/−^* and *Epm2b^−/−^* treated mice, as indicated by a higher discrimination index (Figure 5B and E).

Additionally, we assessed susceptibility to myoclonic jerks following intraperitoneal injection of PTZ at 30 mg/kg, 5 months after rAAVP31‐h*EPM2A* and rAAVP31‐h*EPM2B* treatment (Figure 5C and F). Although the incidence of myoclonic jerks was markedly higher in *Epm2a^−/−^* mice injected with rAAVP31‐Null (64.2%) compared to WT (28.6%), the difference was not statistically significant (Figure 5C). Treatment with rAAVP31‐h*EPM2A* reduced this proportion to 42.1%, showing a clear trend towards improvement, though not reaching statistical significance (Figure 5C). Similarly, *Epm2b^−/−^* mice injected with rAAVP31‐Null showed increased myoclonic jerks (42.85%) compared to WT mice, while rAAVP31‐h*EPM2B* treatment lowered this to 33.3% (Figure 5F), though no significant differences were observed between groups either (Figure 5F).

### 3.3 Electrophysiological improvements in the dentate gyrus and striatum

To explore the mechanisms behind the behavioural improvements, we conducted electrophysiological analyses in the hippocampus and striatum, two key regions involved in seizures and memory processes.

**FIGURE 6:**
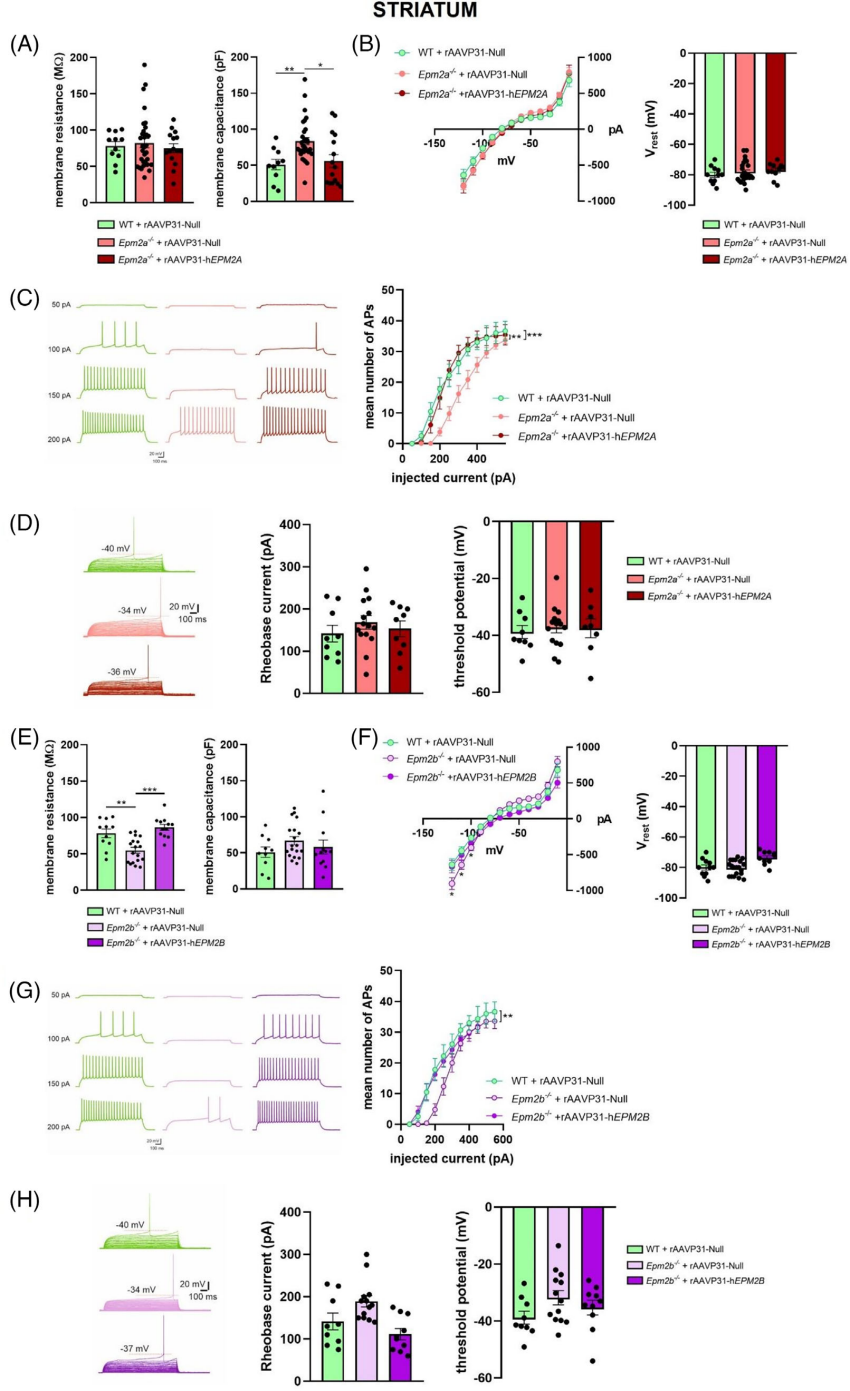
Basal membrane properties and action potential discharges of striatal MSNs in WT, and *Epm2a^−/−^* and *Epm2b^−/−^* mice after IV injection of rAAVP31‐Null, rAAVP31‐h*EPM2A* or rAAVP31‐h*EPM2B* vectors. (A) Left panel: Histograms of membrane resistance of MSNs showing no differences between the three groups (Rm: WT = 78.21 ± 6.0 MΩ, *n* = 11; *Epm2a^−/−^* +rAAVP31‐Null = 82.08 ± 6.5 MΩ, *n* = 32; *Epm2a^−/−^* +rAAVP31‐h*EPM2A *= 75.12 ± 6.1 MΩ, *n* = 15;); right panel: histograms of membrane capacitance showing a significantly increase in MSNs of *Epm2a^−/−^* +rAAVP31‐Null compared to WT. Note that IV injection with rAAVP31‐h*EPM2A* vector completely restored membrane capacitance to control values (Cm: WT = 50.94 ± 7.3 pF, *n* = 10; *Epm2a^−/−^* +rAAVP31‐Null = 83.38 ± 5 pF, *n* = 32; *Epm2a^−/−^* +rAAVP31‐h*EPM2A *= 55.92 ± 8.9 pF, *n* = 16; *p < .05, **p < .01 Student's *t*‐test). (B) Mean current–voltage plot (left) recorded from MSNs of WT, *Epm2a^−/−^* +rAAVP31‐Null and *Epm2a^−/−^* +rAAVP31‐h*EPM2A* mice during injection of hyperpolarising and depolarising current steps, showing no differences between the three groups. On the right, histograms of resting membrane potential show no differences between the three groups (WT = –80 ± 1.7 mV, *n* = 11; *Epm2a^−/−^* +rAAVP31‐Null = –78.1 ± 1.3 mV, *n* = 26; *Epm2a^−/−^* +rAAVP31‐h*EPM2A *= –77.2 ± 1.5 mV, *n* = 11). (C) Action potential (AP) firing patterns in response to 50 pA‐stepped depolarising current injections (1200 ms duration) from MSNs in WT and *Epm2a^−/−^* mice injected with either rAAVP31‐Null or rAAVP31‐h*EPM2A* vectors. The plot shows mean (± SEM) number of MSN APs at the different input currents (*n* = 11 neurons for WT, 22 for *Epm2a^−/−^* +rAAVP31‐Null, 10 for *Epm2a^−/−^* +rAAVP31‐h*EPM2A;* **p < .01 *Epm2a^−/−^* +rAAVP31‐Null vs. *Epm2a^−/−^* +rAAVP31‐h*EPM2A*, ***p < .001 WT vs. *Epm2a^−/−^* +rAAVP31‐Null, two‐way ANOVA). (D) Example of current‐clamp recordings (5 pA‐stepped depolarising current injections; 50 ms), scaled to show the AP threshold, in MSNs of WT, *Epm2a^−/−^* +rAAVP31‐Null and *Epm2a^−/−^* +rAAVP31‐h*EPM2A* mice. The plots show similar rheobase current and AP threshold for the three groups (IRheo: WT = 141.7 ± 19.8 pA, *n* = 9; *Epm2a^−/−^* +rAAVP31‐Null = 168.3 ± 16.1 pA, *n* = 15; *Epm2a^−/−^* +rAAVP31‐h*EPM2A *= 153.3 ± 18.3 pA, *n* = 9; AP threshold: WT = –38.80 ± 2.3 mV, *n* = 9; *Epm2a^−/−^* +rAAVP31‐Null = –37.22 ± 1.9 mV, *n* = 15; *Epm2a^−/−^* +rAAVP31‐h*EPM2A *= –37.55 ± 3.3 mV, *n* = 8). (E) Left panel: histograms of membrane resistance showing a significant reduction in MSNs of *Epm2b^−/−^* +rAAVP31‐Null relative to WT. Note that IV injection with rAAVP31‐h*EPM2B* vector completely restored membrane resistance to control values (Rm: WT = 78.21 ± 6 MΩ, *n* = 11; *Epm2b^−/−^* +rAAVP31‐Null = 54.88 ± 4 MΩ, *n* = 18; *Epm2b^−/−^* +rAAVP31‐h*EPM2B *= 86.54 ± 4.1 MΩ, *n* = 12; **p < .01, ***p < .001 Student's *t*‐test); right panel: histograms of MSNs membrane capacitance showing no differences between the three groups (Cm: WT = 50.94 ± 7.3 pF, *n* = 10; *Epm2b^−/−^* +rAAVP31‐Null = 66.87 ± 5.6 pF, *n* = 19; *Epm2b^−/−^* +rAAVP31‐h*EPM2B *= 58.06 ± 7.3 pF, *n* = 12). (F) Mean current–voltage plot (left) recorded from MSNs of WT, *Epm2b^−/−^* +rAAVP31‐Null and *Epm2b^−/−^* +rAAVP31‐h*EPM2B* mice during injection of hyperpolarising and depolarising current steps, showing a significant increase of the inward currents elicited at hyperpolarised membrane potential in *Epm2b^−/−^* compared to WT, which is completely restored after IV injection of *Epm2b^−/−^* +rAAVP31‐h*EPM2B* vector (–120 mV: WT = –638.9 ± 81.6 pA, *n* = 12; *Epm2b^−/−^* +rAAVP31‐Null = –902.0 ± 82.9 pA, *n* = 21; *Epm2b^−/−^* +rAAVP31‐h*EPM2B *= –666.0 ± 86.1 pA, *n* = 11; –110 mV: WT = –444.2 ± 56.7 pA, *n* = 12; *Epm2b^−/−^* +rAAVP31‐Null = –641.7 ± 61.3 pA, *n* = 21; *Epm2b^−/−^* +rAAVP31‐h*EPM2B *= –501.9 ± 70.8 pA, *n* = 11; –100 mV: WT = –265.4 ± 33.2 pA, *n* = 12; *Epm2b^−/−^* +rAAVP31‐Null = –400.5 ± 42.8 pA, *n* = 21; *Epm2b^−/−^* +rAAVP31‐h*EPM2B *= –342.3 ± 54.8 pA, *n* = 11; WT vs. *Epm2b^−/−^* +rAAVP31‐Null *p < .05, Student's *t*‐test). On the right, histograms of resting membrane potential showing no differences between the three groups (WT = –80.0 ± 1.7 mV, *n* = 11; *Epm2b^−/−^* +rAAVP31‐Null = –80.3 ± 1.1 mV, *n* = 20; *Epm2b^−/−^* +rAAVP31‐h*EPM2B *= –73.7 ± 1.4 mV, *n* = 10). (G) Action potential (AP) firing patterns in response to 50 pA‐stepped depolarising current injections (1200 ms duration) from MSNs in WT and *Epm2b^−/−^* mice injected with either rAAVP31‐Null or rAAVP31‐h*EPM2B* vectors. The plot shows mean (± SEM) number of MSN APs at the different input currents (150 pA: WT = 10.45 ± 2.9, *n* = 11; *Epm2b^−/−^* +rAAVP31‐Null = .4 ± .3, *n* = 20; *Epm2b^−/−^* +rAAVP31‐h*EPM2B *= 10.56 ± 2.6, *n* = 9; 200 pA: WT = 17.82 ± 3.6, *n* = 11; *Epm2b^−/−^* +rAAVP31‐Null = 4.8 ± 1.9, *n* = 20; *Epm2b^−/−^* +rAAVP31‐h*EPM2B *= 16.22 ± 1.8, *n* = 9; **p < .01 WT vs. *Epm2b^−/−^* +rAAVP31‐Null, two‐way ANOVA). (H) Example of current‐clamp recordings (5 pA‐stepped depolarising current injections; 50 ms), scaled to show the AP threshold, in MSNs of WT, *Epm2b^−/−^* +rAAVP31‐Null and *Epm2b^−/−^* +rAAVP31‐h*EPM2B* mice. The plots show an increasing trend of rheobase current and a significant depolarised AP threshold in MSNs of *Epm2b^−/−^* mice relative to WT (IRheo: WT = 141.7 ± 19.8 pA, *n* = 9; *Epm2b^−/−^* +rAAVP31‐Null = 189.2 ± 13.5 pA, *n* = 13; *Epm2b^−/−^* +rAAVP31‐h*EPM2B *= 111.5 ± 13.3 pA, *n* = 10; AP threshold: WT = –38.8 ± 2.3 mV, *n* = 9; *Epm2b^−/−^* +rAAVP31‐Null = –31.78 ± 2.5 mV, *n* = 13; *Epm2b^−/−^* +rAAVP31‐h*EPM2B *= –35.28 ± 2.6 pA, *n* = 10). Data are reported as means ± SEM.

We investigated the effect of gene therapy on the basal membrane properties of striatal MSN. MSNs of *Epm2a^−/−^* mice presented increased membrane capacitance compared to WT (Figure 6A, right panel), while membrane resistance (Figure 6A, left panel), the current‐voltage relationship (Figure 6B, left panel), and the resting membrane potential (V_rest_, Figure 6B) were similar to those of WT mice. Next, we assessed membrane excitability. We found a significant reduction in the number of action potentials (AP) in *Epm2a* ^−/−^ compared to WT mice (Figure 6C). Moreover, we found an increasing trend in the rheobase current, although it was not statistically significant (Figure 6D). The threshold potential was also comparable between WT and *Epm2a* ^−/−^ mice (Figure 6D). Treatment of *Epm2a* ^−/−^ mice with the rAAVP31‐h*EPM2A* vector completely restored MSN membrane capacitance and AP frequency to WT levels.

MSNs from *Epm2b* ***^−^^/^^−^*** mice displayed significantly lower membrane resistance compared to their WT counterpart, while membrane capacitance showed an increasing trend (Figure 6E). Furthermore, the analysis of the current‐voltage relationship revealed a significant increase in inward currents elicited at hyperpolarising voltage steps, while no differences in resting membrane potential were observed between *Epm2b^−/−^* and WT mice (Figure 6F). The number of APs was significantly reduced in *Epm2b* ^−/−^ mice compared to WT mice (Figure 6G). This reduction was particularly evident during the initial steps of depolarising current injection, which triggered significantly fewer action potentials in the *Epm2b* ^−/−^ animals (Figure 6G). We did not observe any significant alterations in either rheobase current or threshold potential compared to WT (Figure 6H). Gene therapy treatment with rAAVP31‐h*EPM2B* completely restored the basal membrane properties of MSNs to physiological levels (Figure 6E–H).

**FIGURE 7:**
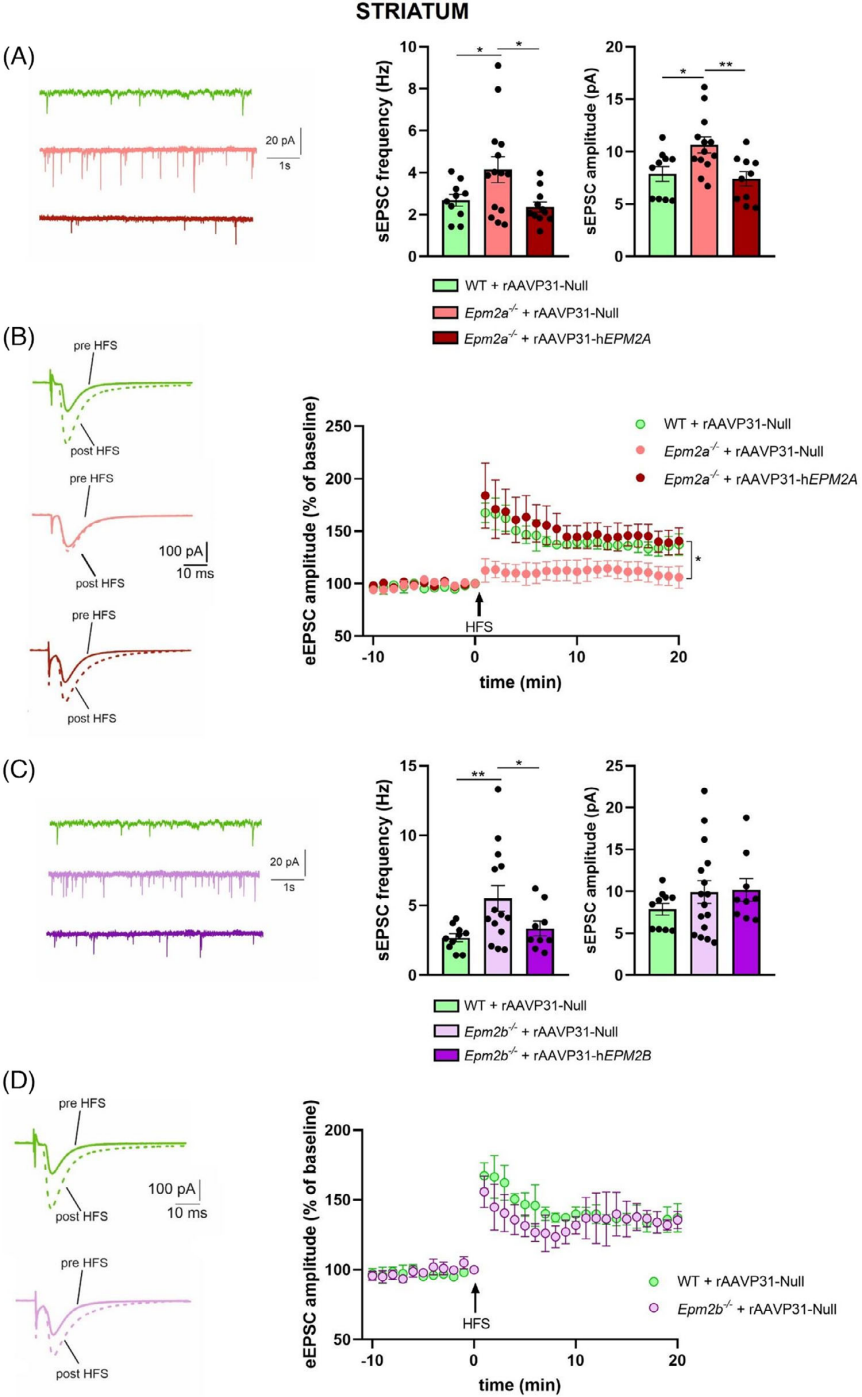
Cortico‐striatal transmission and synaptic plasticity in the nucleus striatum of WT, *Epm2a^−/−^* and *Epm2b^−/−^* mice after IV injection of rAAVP31‐h*EPM2A*, rAAVP31‐h*EPM2B*, or rAAVP31‐Null vectors. (A) Representative sEPSC traces and histograms of the sEPSC mean frequency and amplitude of MSNs recorded from WT (green), *Epm2a^−/−^* +rAAVP31‐Null (pink) and *Epm2a^–/–^* +rAAVP31‐h*EPM2A* (red) mice, showing a significant increase of sEPSC frequency and amplitude in *Epm2a^−/−^* mice with respect to WT. Note the rescue of both sEPSC frequency and amplitude to control levels in mice injected with the rAAVP31‐h*EPM2A* vector (sEPSC frequency: WT = 2.68 ± .3 Hz, *n* = 10; *Epm2a^−/−^* +rAAVP31‐Null = 4.15 ± .6 Hz, *n* = 14; *Epm2a^−/−^* +rAAVP31‐h*EPM2A *= 2.36 ± .2 Hz, *n* = 11; sEPSC amplitude: WT = 7.87 ± .7 pA, *n* = 10; *Epm2a^−/−^* +rAAVP31‐Null = 12.4 ± 1.9 pA, *n* = 14; *Epm2a^−/−^* +rAAVP31‐h*EPM2A *= 7.4 ± .7 pA, *n* = 11; *p < .05, **p < .01 Student's *t*‐test). (B) Representative eEPSC traces and time course plot of eEPSC amplitudes recorded in the MSNs of dorsolateral striatum before and after HFS protocol administration on WT (green), *Epm2a^−/−^* +rAAVP31‐Null (pink) and *Epm2a^−/−^* +rAAVP31‐h*EPM2A* (red) mice brain slices. Note that the IV injection of rAAVP31‐h*EPM2A* vector is able to rescue MSNs LTP to physiological levels (WT: 137 ± 10.2%, *n* = 6; *Epm2a^−/−^* +rAAVP31‐Null:106 ± 10.6%, *n* = 9; *Epm2a^−/−^* +rAAVP31‐h*EPM2A*: 140 ± 12.6%, *n* = 8, *p < .05, two‐way ANOVA). (C) Representative sEPSC traces and histograms of the mean frequency and amplitude of the sEPSC of MSNs recorded from WT (green), *Epm2b^−/−^* +rAAVP31‐Null (light purple) and *Epm2b^–/–^* +rAAVP31‐h*EPM2B* (purple) mice, showing a significant increase in sEPSC frequency and a trend towards reduction of sEPSC amplitude in *Epm2b^−/−^* mice with respect to WT. Note the rescue of sEPSC frequency to control levels in mice injected with the rAAVP31‐h*EPM2B* vector (sEPSC frequency: WT = 2.68 ± .3 Hz, *n* = 10; *Epm2b^−/−^* +rAAVP31‐Null = 5.50 ± .9 Hz, *n* = 14; *Epm2b^−/−^* +rAAVP31‐h*EPM2B *= 3.35 ± .5 Hz, *n* = 9; sEPSC amplitude: WT = 7.87 ± .7 pA, *n* = 10; *Epm2b^−/−^* +rAAVP31‐Null = 9.92 ± 1.3 pA, *n* = 14; *Epm2b^−/−^* +rAAVP31‐h*EPM2B *= 10.18 ± 1.3 pA, *n* = 9; *p < .05, **p < .01 Student's *t*‐test). (D) Representative eEPSC traces and time course plot of eEPSC amplitudes recorded in the MSNs of dorsolateral striatum before and after HFS protocol administration on WT (green), *Epm2b^−/−^* +rAAVP31‐Null (light purple), showing no differences between the two groups (WT: 137 ± 10.2%, *n* = 6; *Epm2b^−/−^* +rAAVP31‐Null: 135 ± 6.1%, *n* = 5). Data are reported as means ± SEM.

To further investigate cortico‐striatal transmission, we examined sEPSCs and LTP of MSNs. In *Epm2a* ^−/−^ mice, we found a significant increase in both sEPSC frequency and amplitude (Figure 7A) compared to WT. The rAAVP31‐h*EPM2A* vector fully restored both sEPSC frequency and amplitude in *Epm2a* ^−/−^ mice (Figure 7A). We then applied an LTP induction protocol to MSNs, as shown in Figure 7B, and we found that *Epm2a^−/−^* MSNs were completely unable to perform LTP. Gene therapy with the rAAVP31‐h*EPM2A* vector was highly effective in fully restoring LTP as well (Figure 7B).

Similar experiments were conducted in *Epm2b* ^−/−^ mice, where we found a significant increase in sEPSC frequency, along with a noticeable, albeit not statistically significant, trend towards increase sEPSC amplitude (Figure 7C). Moreover, LTP of MSNs remained at physiological levels in *Epm2b* ^−/−^ mice (Figure 7D). Gene therapy with the rAAVP31‐h*EPM2B* vector fully normalised sEPSC frequency, although it did not appear to affect sEPSC amplitude.

**FIGURE 8:**
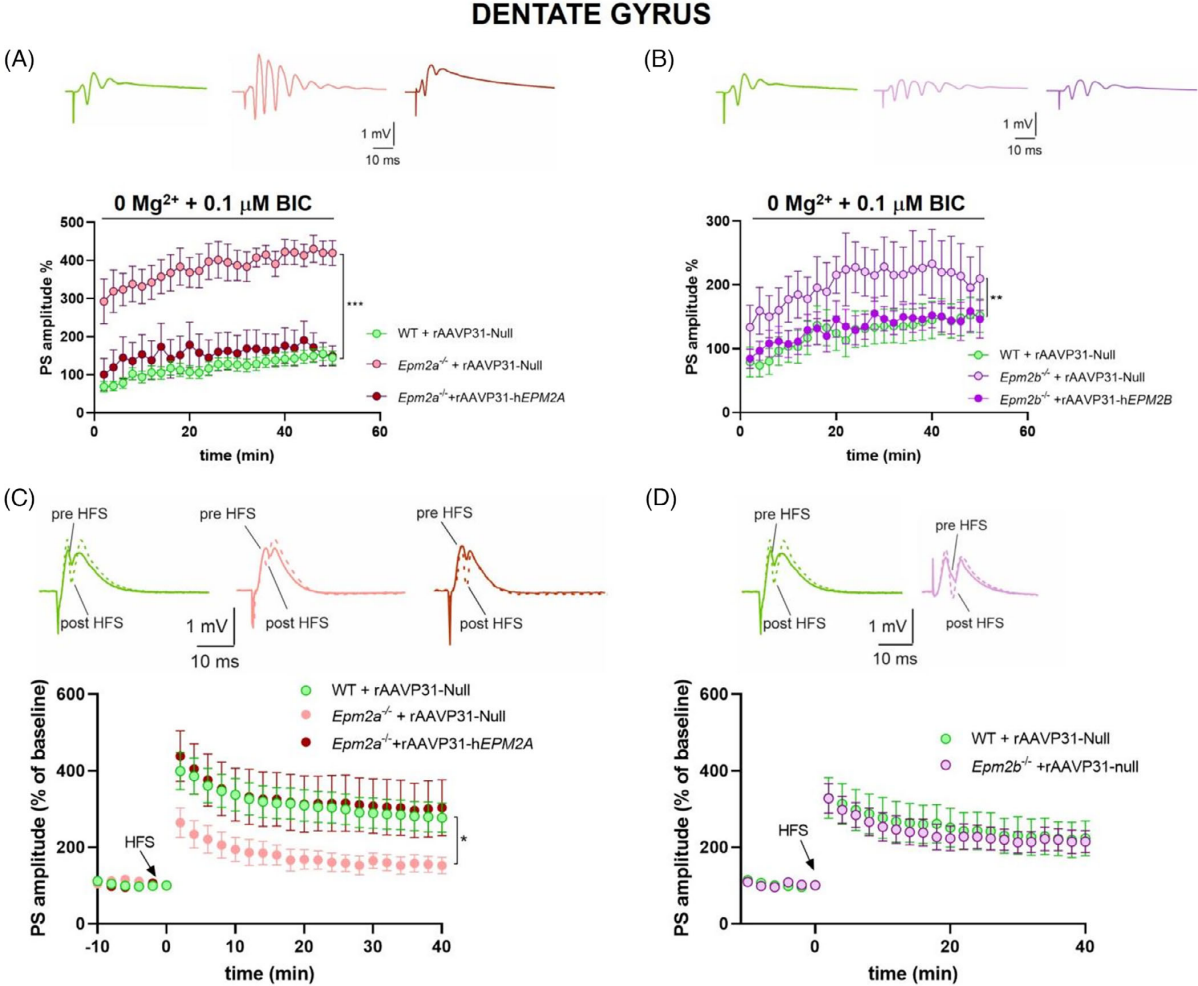
Epileptic‐like activity and LTP in DG slices of WT, *Epm2a^−/−^* and *Epm2b^−/−^* mice after IV injection of rAAVP31‐h*EPM2A*, rAAVP31‐h*EPM2B* or rAAVP31‐Null vectors. (A) Representative traces of FPs and time‐course graph of PS amplitude recorded in the DG of WT+rAAVP31‐Null (green), *Epm2a^−/−^* +rAAVP31‐Null (pink), and *Epm2a^−/−^* +rAAVP31‐h*EPM2A* (red) mice in a magnesium‐free ACSF in the presence of  .1 µM bicuculline, showing a time‐dependent increase of the epileptic‐like activity in *Epm2a^−/−^* +rAAV‐Null mice compared to WT (WT: 145 ± 17.7 %, *n* = 13; *Epm2a^−/−^* +rAAVP31‐Null: 419.8 ± 32.5 %, *n* = 9; ***p < .001 two‐way ANOVA). Note that injection of rAAVP31‐h*EPM2A* vector rescues the epileptic‐like activity to control values (WT: 145 ± 17.7 %, *n* = 13; *Epm2a^−/−^* +rAAV‐hEPM2A: 150 ± 26.4 %, *n* = 9). (B) Representative traces of FPs and time‐course graph of PS amplitude recorded in the DG of WT+rAAVP31‐Null (green), *Epm2b^−/−^* +rAAVP31‐Null (light purple) and *Epm2b^−/−^* +rAAVP31‐h*EPM2B* (purple) mice in a magnesium‐free ACSF in the presence of  .1 µM bicuculline, showing a time‐dependent increase of the epileptic‐like activity in *Epm2b^−/−^* +rAAV‐Null mice relative to WT (WT: 152 ± 26.3 %, *n* = 16; *Epm2b^−/−^* +rAAVP31‐Null: 210 ± 50.2 %, *n* = 6; **p < .01 two‐way ANOVA). Injection of rAAVP31‐h*EPM2B* vector reduces the epileptic‐like activity (WT: 152 ± 26.3 %, *n* = 16; *Epm2b^−/−^* +rAAVP31‐h*EPM2B*: 146 ± 29.7 %, *n* = 10) C) Representative PSs traces recorded before (left) and 40 min after (right) the HFS protocol in DG slices of WT (green), *Epm2a^−/−^* +rAAVP31‐Null (pink) and *Epm2a^−/−^* +rAAVP31‐h*EPM2A* (red) mice. The time‐course plot of PSs amplitudes recorded in the DG before and after HFS protocol shows a significant impairment of LTP in *Epm2a^−/−^* +rAAVP31‐Null mice compared to WT (WT: 277 ± 37.9 %, *n* = 13; *Epm2a^−/−^* +rAAVP31‐Null: 152 ± 21.6 %, *n* = 6; *p < .05 two‐way ANOVA). Note that injection of rAAV‐hEPM2A vector rescues LTP to physiological levels (WT: 277 ± 37.9 %, *n* = 13; *Epm2a^−/−^* +rAAVP31‐h*EPM2A*: 303 ± 73.2 %, *n* = 10). D) Representative PSs traces recorded before (left) and 40 min after (right) the HFS protocol in DG slices of WT (green) and *Epm2b^−/−^* +rAAVP31‐Null (light purple) mice. The time‐course plot of PS amplitudes recorded in the DG before and after HFS protocol shows no differences between the two groups (WT: 223 ± 45.8 %, *n* = 11; *Epm2b^−/−^* +rAAVP31‐Null: 215 ± 28.7 %, *n* = 5). Data are reported as means ± SEM.

Using extracellular recordings of field potentials, we mimicked epileptic‐like activity in vitro, on hippocampal DG slices, by evoking repetitive population spikes^73^ in the presence of  .1 µM bicuculline in an Mg^2+^‐free solution as described previously.^70^, ^74^ Under these experimental conditions, mild epileptic‐like activity was induced in control mice.^70^ Our data showed increased epileptic‐like activity in the DG slices of both *Epm2a^−/−^* and *Epm2b^−/−^* mice compared to WT, as revealed by the elevated PS amplitude (Figure 8A and B). In contrast, the DG of *Epm2b^−/−^* mice displayed a milder epileptic‐like phenotype compared to *Epm2a^−/−^* mice. These findings align with our observations, which indicate that *Epm2a^−/−^* mice exhibit a higher sensitivity to PTZ compared to *Epm2b^−/−^* mice. Systemic delivery of the rAAVP31‐h*EPM2A* vector in *Epm2a^−/−^* mice fully restored epileptic‐like activity to levels observed in WT animals (Figure 8A). In *Epm2b^−/−^* mice, rAAVP31‐h*EPM2B* reduced epileptic‐like activity; however, the PS amplitude in *Epm2b^−/−^* + rAAVP31‐h*EPM2B* mice was not statistically different from either WT mice or *Epm2b^−/−^* + rAAVP31‐Null mice (Figure 8B). We also examined synaptic plasticity by inducing LTP in DG granule cells of both *Epm2a^−/−^* and *Epm2b^−/−^* mice. As shown in Figure 8C, *Epm2a^−/−^* granule cells completely lose their ability to perform LTP, whereas LTP in *Epm2b^−/−^* mouse DG was maintained at physiological levels (Figure 8D). Notably, IV gene therapy with the rAAVP31‐h*EPM2A* vector fully restored LTP in the DG of *Epm2a^−/−^* mice.

### 3.4 Gene therapy decreases LB formation, diminishes astrogliosis, and slightly reduces neurodegeneration

**FIGURE 9:**
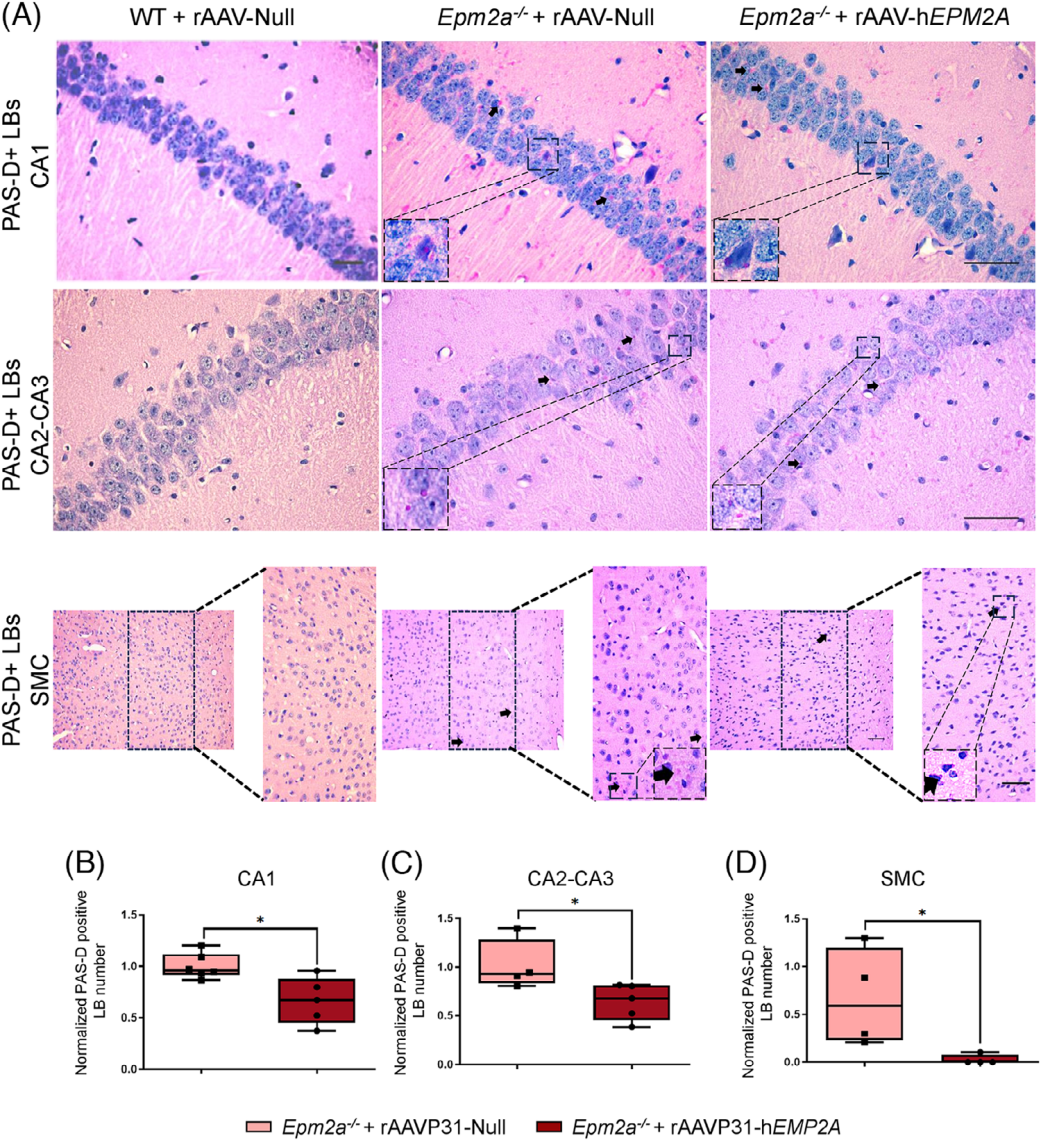
LB formation in hippocampal CA1 and CA2‐CA3 regions, and in the SMC of *Epm2a^−/−^* mice IV injected with rAAVP31‐h*EPM2A*. (A) PAS‐D staining labeling LBs in WT and *Epm2a^−/−^* mice, at 6 months of age, 5 months after treatment with rAAVP31‐h*EPM2A* or rAAVP31‐Null. Scale bar = 50 µm. (B‐D) Quantitative comparison of LB numbers in (B) CA1 (*Epm2a^−/−^* +rAAVP31‐Null vs. *Epm2a^−/−^* +rAAVP31‐h*EPM2A p*‐value = .0303), (C) CA2 (*Epm2a^−/−^+*rAAVP31‐Null vs. *Epm2a^−/−^+*rAAVP31‐h*EPM2A p*‐value = .0397) and (D) SMC (*Epm2a^−/−^+*rAAVP31‐Null vs. *Epm2a^−/−^+*rAAVP31‐h*EPM2A p*‐value = .0286). In the SMC, the quantitative analysis was carried out in the enlarged region (width: 747 px; height: 1550 px), corresponding to layers IV‐V of the sensorimotor cortex. Data are shown as the median of independent samples. The bars in the box plots show the minimum and maximum values. Values were normalised using *Epm2a^−/−^* mice treated with rAAVP31‐Null values. A non‐parametric Mann–Whitney test was performed for statistical analysis. *p < .05. *n* = 4–6 mice per group.

**FIGURE 10:**
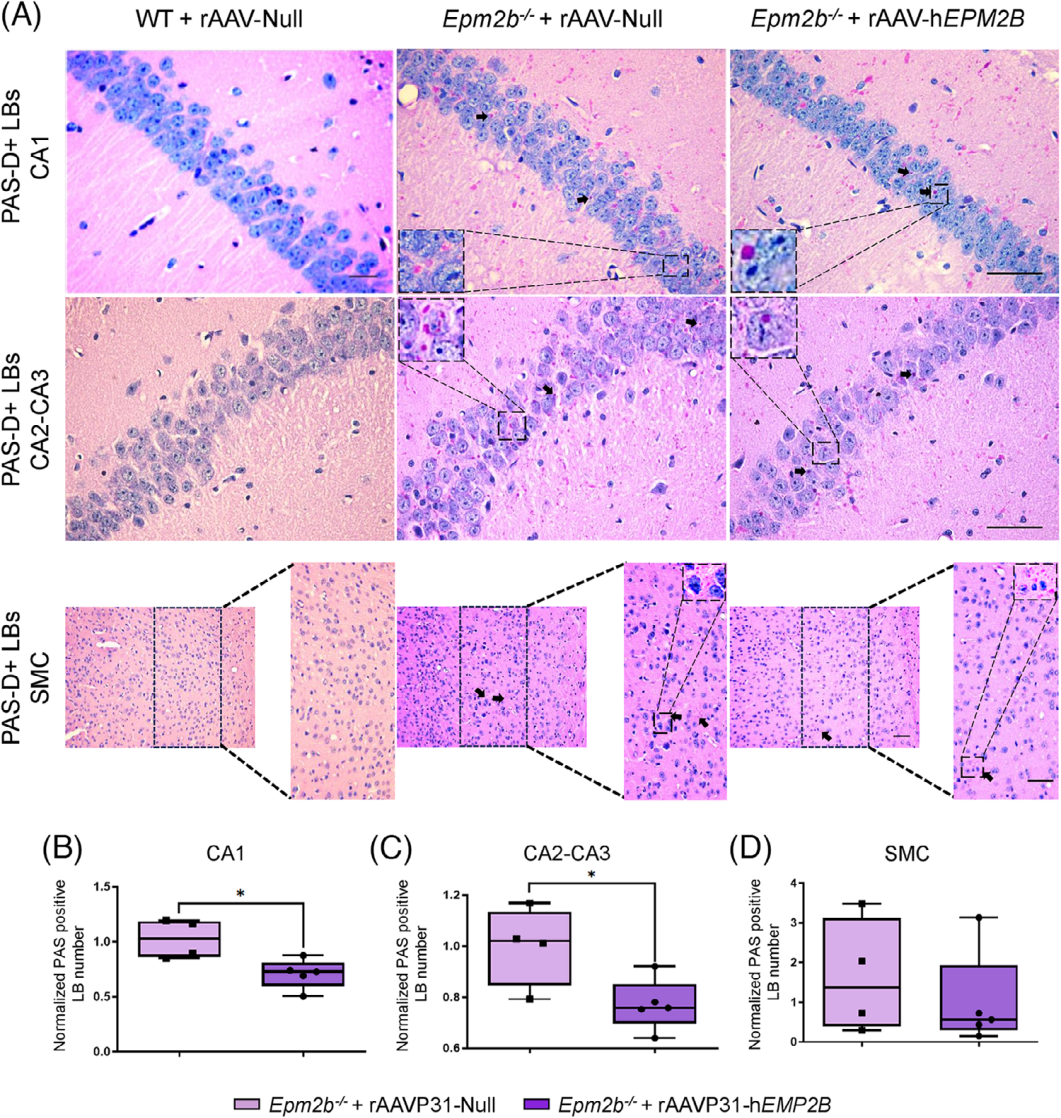
LB formation in the CA1 and CA2‐CA3 regions of the hippocampus, and in the SMC of *Epm2b^−/−^* mice after IV injection of rAAVP31‐h*EPM2B*. (A) PAS‐D staining to visualise LBs in WT and *Epm2b^−/−^* 6‐month‐old mice, 5 months after rAAVP31‐h*EPM2B* or rAAVP31‐Null treatment. Scale bar = 50 µm. (B–D) Quantitative comparison of LB numbers in (B) CA1 (*Epm2b^−/−^* +rAAVP31‐Null vs. *Epm2b^−/−^* +rAAVP31‐h*EPM2B p*‐value = .0317), (C) CA2 (*Epm2b^−/−^* +rAAVP31‐Null vs. *Epm2b^−/−^* +rAAVP31‐h*EPM2B p*‐value = .0317) and (D) SMC (*Epm2b^−/−^* +rAAVP31‐Null vs. *Epm2b^−/−^* +rAAVP31‐h*EPM2B p*‐value = .4524). The quantitative analysis in the SMC was carried out in the enlarged region (width: 747 px; height: 1550 px), corresponding to layers IV‐V of the sensorimotor cortex. Data are shown as the median of independent samples. Boxplot bars represent the minimum and maximum values. Values were normalised using *Epm2b^−/−^* mice treated with rAAVP31‐Null values. Statistical analysis was performed using a non‐parametric Mann–Whitney test. *p < .05. *n* = 4–6 mice per group.

We analysed the number of LBs in the Cornu Ammonis (CA) 1 and CA2‐CA3 regions of the hippocampus, as well as in layers IV‐V of the sensorimotor cortex 5 months after treatment administration. A significant reduction in the number of LB was observed in the CA1 (Figures 9A and B and 10A and B) and CA2‐CA3 regions (Figures 9A and C and 10A and C) of the hippocampus in both *Epm2a* ***^−^^/^^−^*** and *Epm2b^−/−^* mice following treatment, compared to those receiving the rAAVP31‐Null vector. Furthermore, a significant decrease in the number of LBs was detected in layers IV‐V of the sensorimotor cortex of treated *Epm2a* ***^−^^/^^−^*** mice (Figure 9A and D). This effect was not observed in the corresponding cortical layers of treated *Epm2b* ***^−^^/^^−^*** mice (Figure 10A and D).

The effect of early systemic administration of the therapeutic vectors on neuroinflammation in *Epm2a* ***^−^^/^^−^*** (Figure S1A) and *Epm2b* ***^−^^/^^−^*** (Figure S2A) mice was assessed 5 months after IV injections using a GFAP antibody. Quantification of GFAP‐positive cells in the CA1 field of the hippocampus revealed a reduction in GFAP‐immunostained cells in treated *Epm2a^−/−^* (Figure S1A and B) and *Epm2b^−/−^* (Figure S2A and B) mice compared to those treated with the Null vector, which showed significantly increased astrogliosis relative to control mice. Astrogliosis was further examined in the CA2‐CA3 regions of the hippocampus (Figures S1A and C and S2A and C) and layers IV‐V of sensorimotor cortex (Figures S1A and D and S2A and D). A reduction in astrogliosis was observed in the CA2‐CA3 regions of treated *Epm2b^−/−^* mice compared to those receiving the Null vector (Figure S2A and C), which, as in CA1, exhibited a significant increase in reactive astrocytes. However, in layers IV‐V of the sensorimotor cortex, no significant differences were noted (Figure S2A and D). In *Epm2a^−/−^* mice no significant differences in astrogliosis were detected, either in the CA2‐CA3 region of the hippocampus (Figure S1A and C) or in layers IV‐V of the sensorimotor cortex (Figure S1A and D).

The effect of the therapeutic vectors on neurodegeneration was examined using NeuN immunostaining 5 months post‐IV injection (Figures S3 and S4). A slight trend towards a decrease in NeuN‐positive cells was observed in the CA1, CA2‐CA3, and layers IV‐V of the sensorimotor cortex of *Epm2a^−/−^* and *Epm2b^−/−^* mice injected with the rAAVP31‐Null vector, though these differences were not statistically significant compared to WT mice (Figures S3A–D and S4A–D). Notably, this trend was absent in *Epm2a^−/−^* and *Epm2b^−/−^* mice treated with the therapeutic vectors, where NeuN‐positive cell counts were restored to levels comparable to WT (Figures S3A–D and S4A–D).

### 3.5 Effects of systemically administered viral vectors on liver function and the immune system

**FIGURE 11:**
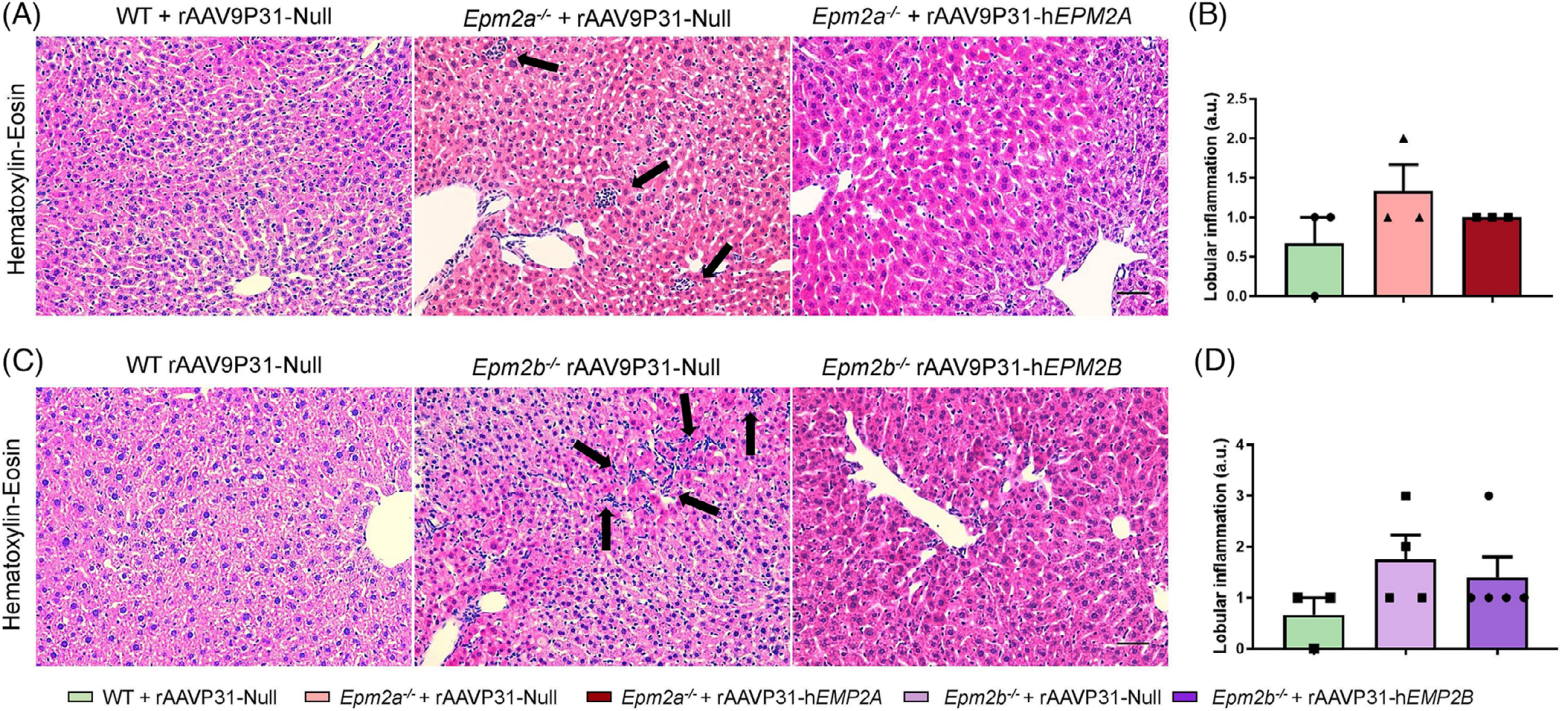
Hepatotoxicity study in 6‐month‐old WT, *Epm2a^−/−^* and *Epm2b^−/−^* mice, 5 months following IV administration of rAAVP31‐h*EPM2A*, rAAVP31‐h*EPM2B* or rAAVP31‐Null vectors. (A, C) H&E staining in liver sections from WT, *Epm2a^−/−^* and *Epm2b^−/−^* mice. (B, D) Semiquantitative analysis of lobular inflammation according to NASH activity scoring criteria. a.u. = arbitrary units. *n* = 3–5 mice per group. Scale bar = 50 µm.

We examined potential liver‐related adverse effects, specifically lobular inflammation and hepatic steatosis in WT, *Epm2a^−/−^* and *Epm2b^−/−^* mice, 5 months after IV injection of the rAAVP31‐Null, rAAVP31‐h*EPM2A*, or rAAVP31‐h*EPM2B* vectors, using H&E staining. No evidence of lobular inflammation was detected in any of the experimental groups (Figure 11A–D). However, a slight, though not statistically significant, increase in lobular inflammation was noted in *Epm2a^−/−^* and *Epm2b^−/−^* mice treated with the rAAVP31‐Null vector compared to WT control mice (Figure 11A–D), suggesting potential liver alterations in these models. Additionally, we found no evidence of steatosis or hepatocellular hypertrophy (Figure 11A and C). Nonetheless, more in‐depth studies are required to evaluate possible hepatic abnormalities in these models.

We also analysed biochemical parameters in the serum of mice 15 months after vectors administration to evaluate potential long‐term liver damage or immune responses (Figure S5A and B). *Epm2b^−/−^* mice exhibited significantly lower glucose levels compared to WT mice. Treatment with the rAAVP31‐h*EPM2B* vector resulted in a normalisation of glucose levels (Figure S5B). Liver function was assessed by measuring levels of the ALP, AST and ALT enzymes, which are commonly used indicators of hepatic health.^75^ Neither vector treatment caused alterations in the levels of the hepatic enzymes AST and ALT. However, IV administration of the rAAVP31‐h*EPM2A* vector led to an increase in ALP and a decrease in glucose (Figure S5A). Considering the histological findings and the normal AST and ALT levels, the observed increase in ALP does not appear to be due to hepatic damage and may instead be associated to other biochemical process.

Finally, to assess potential immune activation, we measured serum levels of IgG and IgM. No significant differences were observed between experimental groups (Figure S5).

## 4 DISCUSSION

A major challenge for the clinical application of gene therapy in CNS diseases lies in the limited ability of viral vectors to effectively reach the brain or spinal cord. The BBB is a significant obstacle to delivering genes into brain tissue after IV administration. Even the most effective natural serotypes, such as AAV9, show limited brain distribution.^59^, ^76^ Various administration routes have been explored over years to target the CNS, including ICV, intraparenchymal or intrathecal injections. While these methods help bypass the BBB, they often provide limited distribution level and transduction capacity.^77^ High‐throughput mutagenesis and directed evolution of viral capsids have expanded the diversity of AAV vectors, leading to the development of serotypes with enhanced ability to cross the BBB.^59^, ^60^, ^63^ Among these variants, the AAV9P31, derived from AAV9, has shown enhanced efficiency for CNS delivery.^59^, ^63^ This variant includes the WPTSYDA sequence insertion between amino acids Q588 and A589 in the variable region VIII (VR‐VIII) of the VP3 capsid protein, resulting in up to a 385‐fold increase in CNS tropism compared to wild‐type AAV9.^59^, ^63^ However, recent studies have highlighted important limitations in the ability of AAV9P31 to cross BBB in non‐human primates and rats.^61^ While cross‐species variability in receptor usage remains a critical barrier in the development of clinically translatable AAV vectors, recent studies have identified capsids that interact with conserved receptors – such as the VCAP‐102 capsid, which targets the ALP receptor and enables efficient CNS delivery in both rodents and primates^62^ – suggesting that the field is moving towards more universal and broadly applicable solutions. Although the 9P31 serotype may not be suitable for clinical gene delivery to the human brain via intravenous administration, it remains a highly valuable tool for basic and preclinical research, particularly in murine models of neurological disorders. Moreover, our data further support its utility in juvenile mice, where systemic delivery achieved rapid and widespread brain transduction, which is especially relevant for treating early‐onset diseases like Lafora disease.

We previously showed that ICV administration of rAAV9‐h*EPM2A* or rAAV9‐h*EPM2B* vectors in 3‐month‐old *Epm2a* ***^−^^/^^−^*** ^58^ or *Epm2b^−/−^* (under review) mice reversed most of the neurological and histopathological symptoms in treated mice. To improve transduction efficiency, expand biodistribution, and support clinical translation, we evaluated the therapeutic potential of IV administration of the rAAVP31 serotype carrying the human *EPM2A* or *EPM2B* genes. Early diagnosis and treatment are especially important in neurodegenerative diseases. Previous studies in our laboratory have shown that early treatment with metformin was more effective in delaying disease progression when administered during the initial stages of the disease.^48^ Therefore, we evaluated the effect of IV administration of these vectors in 1‐month‐old *Epm2a^−/−^* or *Epm2b^−/−^* mice. In some assays, these knockout models showed values similar to wild‐type controls, indicating they may not fully capture all aspects of the disease phenotype. Nonetheless, they preserve key pathological, electrophysiological, and behavioural features that are critical for evaluating therapeutic efficacy.

Using the *GFP* reporter gene, we observed that the rAAVP31 vector enabled widespread cellular transduction throughout the CNS, primarily in neurons, and peripheral organs. This highlights the vector's ability to cross the BBB and target tissues beyond the nervous system. Quantification of the transgene expression showed substantial overexpression in various CNS regions compared to endogenous levels of the orthologous genes in WT mice, especially for the h*EPM2B* transgene, aligning with the increased CNS tropism conferred by the VP3 capsid protein modification.^59^, ^60^ We also confirmed translation of the therapeutic genes in their respective proteins by detecting the HA‐tagged proteins, consistent with the broad distribution of the transgenes across multiple CNS regions. Additionally, the vectors achieved comparable transgene expression in the liver and heart, though no expression was found in the quadriceps femoris of *Epm2a^−/−^* and *Epm2b^−/−^* mice. Given that liver and heart tissue damage has been observed in some cases of patients with Lafora disease,^78^, ^79^, ^80^ targeting these tissues with therapeutic vectors may offer potential benefits.

Previous studies from our laboratory showed that restoring malin function via ICV gene therapy in *Epm2b^−/−^* mice at 3 months of age failed to slow LB formation (under review). In our analysis of these aberrant aggregates in *Epm2b^−/−^* mice IV treated with rAAVP31‐h*EPM2B* at early stages, we observed a significant reduction in the number of LBs in the CA1 and CA2‐CA3 regions of the hippocampus, 5 months after IV administration. Furthermore, the rAAVP31‐h*EPM2A* vector effectively decreased the formation of these aggregates in layers IV‐V of the sensorimotor cortex in *Epm2a^−/−^* mice. These findings indicate that early systemic administration of rAAVP31‐h*EPM2A* and rAAVP31‐h*EPM2B* is more effective in regulating glycogen metabolism alterations compared to ICV administration of the rAAV‐h*EPM2A* and rAAV‐h*EPM2B* vectors at 3 months of age.^58^ We hypothesised that the lack of efficacy of ICV treatment in *Epm2b^−/−^* mice at 3 months of age was due to the inability to form the laforin–malin complex, which is essential for interacting with enzymes that regulate glycogen metabolism.^14^ This failure likely resulted from laforin being trapped within the LBs in 3‐month‐old *Epm2b* mice,^29^ rendering malin administration insufficient to halt the formation of LBs.^14^ In contrast, *Epm2b^−/−^* mice at 1 month of age exhibit very few LBs, which may allow for a greater proportion of free, active laforin compared to older mice.^29^ The effectiveness of the therapy in preventing LB formation at 1 month of age supports this hypothesis.

Multiple studies have reported prominent glial activation as a hallmark of disease pathology.^33^, ^36^, ^37^, ^38^, ^39^, ^40^, ^41^, ^42^, ^43^ The rAAVP31‐h*EPM2A* and rAAVP31‐h*EPM2B* therapy led to a significant decrease in astrogliosis in the CA1 region of the hippocampus in both *Epm2a^−/−^* and *Epm2b^−/−^* mice. Additionally, the rAAVP31‐h*EPM2B* vector also reduced astrogliosis in the CA2‐CA3 region of the hippocampus in *Epm2b^−/−^* mice. In layers IV‐V of the sensorimotor cortex of the *Epm2a^−/−^* and *Epm2b^−/−^* mice, we found no significant increase in reactive astrocytes compared to WT mice. Furthermore, we did not observe neurodegeneration in our models at that age relative to control mice. However, there was a trend towards a reduction in the number of NeuN‐positive cells across all analysed regions in *Epm2a^−/−^* mice injected with the rAAVP31‐Null vector, which appeared to return to control levels following treatment with the rAAVP31‐h*EPM2A* vector.

Behavioural experiments also demonstrated a beneficial effect of the treatment on neurological alterations in our models. Additionally, IV gene therapy also reduced the number of myoclonic jerks induced by the epileptogenic agent PTZ. These findings suggest that both vectors hold therapeutic potential to reverse the neurological abnormalities in murine models of Lafora disease. This therapeutic effect is further supported by electrophysiological investigations demonstrating that IV gene therapy preserves normal neuronal functioning while restoring neuronal and network alterations observed in the DG and striatum of these models.

In the striatum of both *Epm2a^−/−^* and *Epm2b^−/−^* mice, we observed alterations in the intrinsic membrane properties of MSNs, such as membrane capacitance and resistance, which are closely linked to changes in the time constant tau and neuronal excitability.^81^, ^82^ Additionally, we found a significant reduction in AP discharge in GABAergic MSNs in both *Epm2a^−/−^* and *Epm2b^−/−^* mice compared to WT mice. This reduction may lead to aberrant striatal output and impaired control of basal ganglia function. These findings collectively suggest substantial alterations in key conductance that disrupt AP homeostasis and the state transitions,^83^ which are essential for action potential firing in the MSNs of Lafora disease mice, warranting further investigation. Gene therapy successfully restored these deficits to control levels.

In line with motor coordination deficits observed in these mice, and consistent with findings from other epilepsy and neurodegenerative disease models,^84^, ^85^ our electrophysiological data reveal altered cortico‐striatal transmission in the dorso‐lateral striatum of both *Epm2a^−/−^* and *Epm2b^−/−^* mice, characterised by a significant increase in sEPSC frequency. In the *Epm2a^−/−^* model, this presynaptic change was accompanied by an increase in sEPSC amplitude. Typically, differences in spontaneous event frequency reflect presynaptic dysfunction, possibly due to hyperexcitable glutamatergic cortical neurons projecting to the striatum, whereas changes in amplitude may indicate postsynaptic alterations, potentially related to glutamatergic receptor dysfunction. Given that, under our experimental conditions, the recorded currents are primarily α‐amino‐3‐hydroxy‐5‐methyl‐4‐isoxazolepropionic acid (AMPA)‐mediated, the increased amplitude in *Epm2a* ^−/−^ mice may result from overexpression or overactivity of AMPA receptors, or from enhanced receptor membrane exposure. These hypothesis is supported by previous studies conducted by our group.^58^ However, further studies are needed to clarify this mechanism.

Additionally, we observed impaired LTP in the dorso‐lateral striatum of *Epm2a^−/−^* mice, whereas LTP was preserved in *Epm2b^−/−^* mice, which display a milder electrophysiological phenotype. Notably, IV gene therapy in these mouse models restored both striatal sEPSC frequency and LTP to physiological levels. While the hippocampus and striatum are traditionally associated with cognitive^86^ and procedural memory,^87^ respectively, recent studies in animal and human models suggest that these memory systems can operate in parallel within a dynamic network.^88^ This hippocampal‐striatal cross‐talk is affected in several neurological disorders, including epilepsy, potentially leading to an imbalance between two memory systems,^88^ an imbalance that may also be present in *Epm2a^−/−^* mice.

Consistent with our previous findings,^58^ we also observed increased epileptic‐like activity and impaired LTP in the DG of *Epm2a^−/−^* mice. In *Epm2b^−/−^* mice, our data confirmed a milder electrophysiological phenotype also in the DG, with lower epileptic‐like activity than in *Epm2a^−/−^* mice, although still significantly elevated compared to WT mice. LTP remained unaffected in the DG of *Epm2b^−/−^* mice. Treatment effectively reduced epileptic‐like activity in both models and restored LTP to control levels in *Epm2a^−/−^* mice.

Hepatotoxicity and immunogenicity are major concerns following systemic administration of AAV vectors.^89^, ^90^ Previous clinical and animal studies have reported hepatic alterations, including inflammation and elevated liver enzymes, following AAV treatment.^91^, ^92^, ^93^, ^94^ In this study, we assessed potential hepatotoxic effects at 5 and 15 months following intravenous treatment administration. No lobular inflammation, ALT/AST elevation, steatosis, or hepatocyte hypertrophy was observed in treated or untreated WT, *Epm2a^−/−^* or *Epm2b^−/−^* mice, except for mild lobular inflammation in *Epm2a^−/−^* and *Epm2b^−/−^* mice injected with the rAAVP31‐Null vector. This suggests hepatic alterations, may be linked to hepatic LBs (also referred as ‘ground glass’ in the liver) noted in both mouse models and patients.^79^, ^95^, ^96^ Elevated ALP levels in *Epm2a^−/−^* mice treated with rAAVP31‐h*EPM2A* were likely unrelated to liver damage, given the normal AST/ALT levels and histological findings. Overall, our analyses did not reveal signs of hepatotoxicity in *Epm2a^−/−^* and *Epm2b^−/−^* mice. Fifteen months after IV treatment, no increase in IgG or IgM levels were detected, suggesting no evident activation of the adaptative immune response under the conditions tested. Further research is needed to evaluate long‐term effects of extended AAV exposure on hepatotoxicity and immunogenicity.

In this study, we evaluated a gene therapy approach through IV administration. When considering our previous work using ICV delivery,^58^ the current findings support IV administration as an alternative strategy, providing a less invasive route that may broaden the therapeutic possibilities, particularly when applied at early disease stages.

## 5 CONCLUSIONS

Gene therapy with the rAAVP31‐h*EPM2A* and rAAVP31‐h*EPM2B* vectors normalises cellular and circuit‐level activity, rescues memory deficits, reduces glial activation and LB formation, and shows a trend towards improved motor coordination, demonstrating broad therapeutic effects. While not all histological and behavioural assays reached statistical significance, we consider these findings to be strong indicators of therapeutic potential and likely predictors of clinical benefit.

No hepatotoxic effects or long‐term activation of the adaptive immune system were detected. These findings support the safety and efficacy of this IV gene therapy approach and underscore its strong potential for clinical translation.

## AUTHOR CONTRIBUTIONS

L.Z.P: conceptualisation, data curation, formal analysis, investigation, methodology, validation, visualisation, writing – original draft, and writing – review & editing; N.I.C: investigation, and methodology; M.S: data curation, formal analysis, investigation, methodology, and writing – original draft; L.B.: data curation, investigation, and methodology; J.C.: data curation, investigation and methodology; G.S.M.: methodology; C.C: formal analysis, funding acquisition, investigation, supervision, validation, visualisation, writing – original draft, and writing – review & editing; M.P.S: conceptualisation, formal analysis, funding acquisition, investigation, project administration, supervision, validation, visualisation, writing – original draft, and writing – review & editing; J.M.S: conceptualisation, formal analysis, funding acquisition, investigation, project administration, supervision, validation, visualisation, writing – original draft, and writing – review & editing.

## CONFLICT OF INTEREST STATEMENT

The authors declare no conflicts of interest.

## ETHICS STATEMENT

The protocols adhere to the guidelines outlined in the ‘Principles of Laboratory Animal Care’ (NIH publication No. 86–23, revised 1985), as well as the European Communities Council Directive (2010/63/EU). They received approval from the Ethical Review Board of the Instituto de Investigación Sanitaria‐Fundación Jiménez Díaz, and the Animal Care and Use Committee at the University of Perugia (authorisation no. 2B818.N.9JX). The ARRIVE guidelines from the National Centre for the Replacement, Refinement & Reduction of Animals in Research were strictly followed.^66^

## Supporting information



Supporting Information

## Data Availability

Data supporting the findings of this study are available from the corresponding author.
