# Supplementary material for: Advances in gene therapy for Lafora disease: Intravenous recombinant adeno‐associated virus‐mediated delivery of EPM2A and EPM2B genes
Source: Clin Transl Med. 2025 Oct 30;15(11):e70514. doi: 10.1002/ctm2.70514 (PMC12576012; doi:10.1002/ctm2.70514)
Supplement: Supplementary file 1 — Supporting Information [file CTM2-15-e70514-s001.docx]

**Supplementary Information**

**Advances in gene therapy for Lafora disease: Intravenous recombinant adeno-associated virus-mediated delivery of *EPM2A* and *EPM2B* genes**

Luis Zafra-Puerta^1,2,3^, Nerea Iglesias-Cabeza^1^, Miriam Sciaccaluga^3,4^, Laura Bellingacci^5^, Jacopo Canonichesi^5^, Gema Sánchez-Martín^1^, Cinzia Costa^7^, Marina P. Sánchez^1*^, José M. Serratosa^1*^

^1^Laboratory of Neurology, Instituto de Investigación Sanitaria-Fundación Jiménez Díaz, Universidad Autónoma de Madrid (IIS-FJD, UAM), 28040 Madrid, Spain

^2^PhD Program in Neuroscience, Universidad Autónoma de Madrid-Cajal Institute, Madrid, Spain 28029

^3^Fondazione Malattie Rare Mauro Baschirotto BIRD Onlus, Longare (VI), Italy.

^4^Department of Life Science, Health, and Health Professions, Link University, Rome, Italy

^5^Section of Physiology, Department of Medicine and Surgery, University of Perugia, Perugia 06132, Italy

^6^Section of Neurophysiopathology, S.M. della Misericordia Hospital, Laboratory of Experimental Neurology, Department of Medicine and Surgery, University of Perugia, Perugia 06132, Italy

* Corresponding authors: José M. Serratosa, MD, PhD / Marina P. Sánchez, PhD

Avda. Reyes Católicos, 2 28040 Madrid, Spain

Phone: 34-91-5504800 Ext. 3251; fax: 34-91-5497700

[joseserratosa@me.com](mailto:joseserratosa@me.com) / msanchezg@fjd.es

**
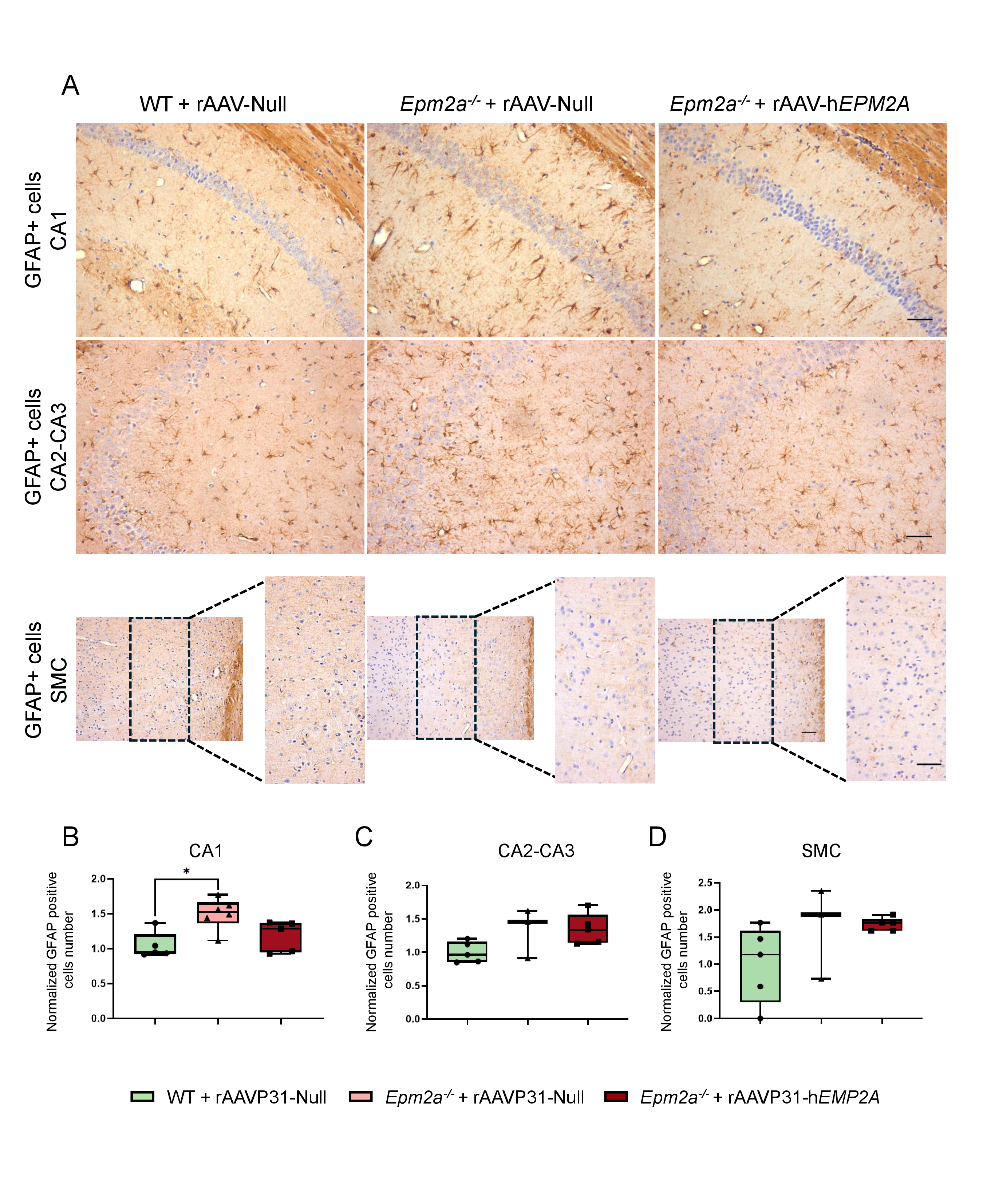
**

**Supplemental Figure 1.** **Quantification of GFAP-positive cells in the CA1 and CA2-CA3 region of the hippocampus and in the SMC of *Epm2a^-/-^* mice IV injected with rAAVP31-h*EPM2A*.** (A) IHC with an anti-GFAP antibody staining reactive astrocytes in WT and *Epm2a^-/-^* mice at 6 months of age, 5 months after rAAVP31-h*EPM2A* or rAAVP31-Null treatment. Scale bar = 50 µm. (B-D) Quantification of reactive astrocytes in (B) CA1 (WT+rAAVP31-Null vs *Epm2a^-/-^*+rAAVP31-Null *p*-Value=0.0160; WT+rAAVP31-Null vs *Epm2a^-/-^*+rAAVP31-h*EPM2A* *p*-Value=>0.9999; *Epm2a^-/-^*+rAAVP31-Null vs *Epm2a^-/-^*+rAAVP31-h*EPM2A* *p*-Value=0.1291), (C) CA2 (WT+rAAVP31-Null vs *Epm2a^-/-^*+rAAVP31-Null *p*-Value=0.3025; WT+rAAVP31-Null vs *Epm2a^-/-^*+rAAVP31-h*EPM2A* *p*-Value=0.1271; *Epm2a^-/-^*+rAAVP31-Null vs *Epm2a^-/-^*+rAAVP31-h*EPM2A* *p*-Value=>0.9999), and (D) SMC (WT+rAAVP31-Null vs *Epm2a^-/-^*+rAAVP31-Null *p*-Value=0.2348; WT+rAAVP31-Null vs *Epm2a^-/-^*+rAAVP31-h*EPM2A* *p*-Value=>0.2350; *Epm2a^-/-^*+rAAVP31-Null vs *Epm2a^-/-^*+rAAVP31-h*EPM2A* *p*-Value=>0.9999). In the SMC, the quantitative analysis was carried out in the enlarged region (width: 747 px; height: 1550 px), corresponding to layers IV-V of the sensorimotor cortex. Data are shown as the median of independent samples. The bars in the box plots show the minimum and maximum values. Values were normalized using WT mice treated with rAAVP31-Null values. A non-parametric Kruskal-Wallis test was performed followed by Dunn´s multiple comparisons. * p < 0.05. n = 3-6 mice per group.

**
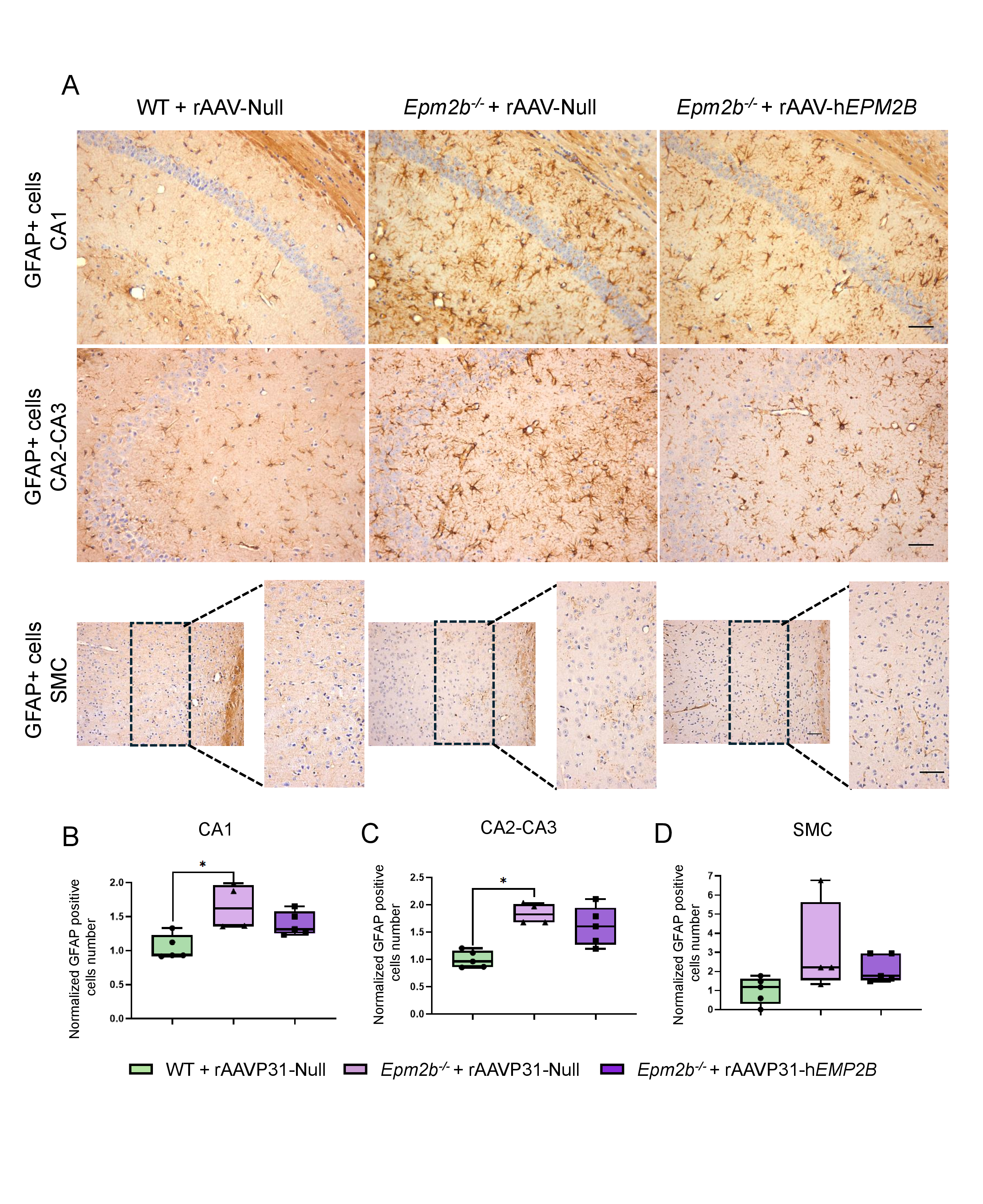
**

**Supplemental Figure 2.** **Quantification of GFAP-positive cells in the CA1 and CA2-CA3 region of the hippocampus and in the SMC of *Epm2b^-/-^* mice IV injected with rAAVP31-h*EPM2B*.** (A) IHC was performed with an anti-GFAP antibody to stain reactive astrocytes in WT and *Epm2b^-/-^* mice 5 months after rAAVP31-h*EPM2B* or rAAVP31-Null treatment. Scale bar = 50 µm. (B-D) Quantification of reactive astrocytes in (B) CA1(WT+rAAVP31-Null vs *Epm2b^-/-^*+rAAVP31-Null *p*-Value=0.0145; WT+rAAVP31-Null vs *Epm2b^-/-^*+rAAVP31-h*EPM2B* *p*-Value=0.2453; *Epm2b^-/-^*+rAAVP31-Null vs *Epm2b^-/-^*+rAAVP31-h*EPM2B* *p*-Value=0.7173), (C) CA2 (WT+rAAVP31-Null vs *Epm2b^-/-^*+rAAVP31-Null *p*-Value=0.0162; WT+rAAVP31-Null vs *Epm2b^-/-^*+rAAVP31-h*EPM2B* *p*-Value=0.0846; *Epm2b^-/-^*+rAAVP31-Null vs *Epm2b^-/-^*+rAAVP31-h*EPM2B* *p*-Value=>0.9999), and (D) SMC (WT+rAAVP31-Null vs *Epm2b^-/-^*+rAAVP31-Null *p*-Value=0.1188; WT+rAAVP31-Null vs *Epm2b^-/-^*+rAAVP31-h*EPM2B* *p*-Value=>0.1451; *Epm2b^-/-^*+rAAVP31-Null vs *Epm2b^-/-^*+rAAVP31-h*EPM2B* *p*-Value=>0.9999). In the SMC, the quantitative analysis was carried out in the enlarged region (width: 747 px; height: 1550 px), corresponding to layers IV-V of the sensorimotor cortex. Data are shown as the median of independent samples, normalized to WT mice treated with rAAVP31-Null values. The bars in the box plots show the minimum and maximum values. Statistical analysis was performed using a non-parametric Kruskal-Wallis test followed by Dunn´s multiple comparisons. * p < 0.05. n = 4-5 mice per group.


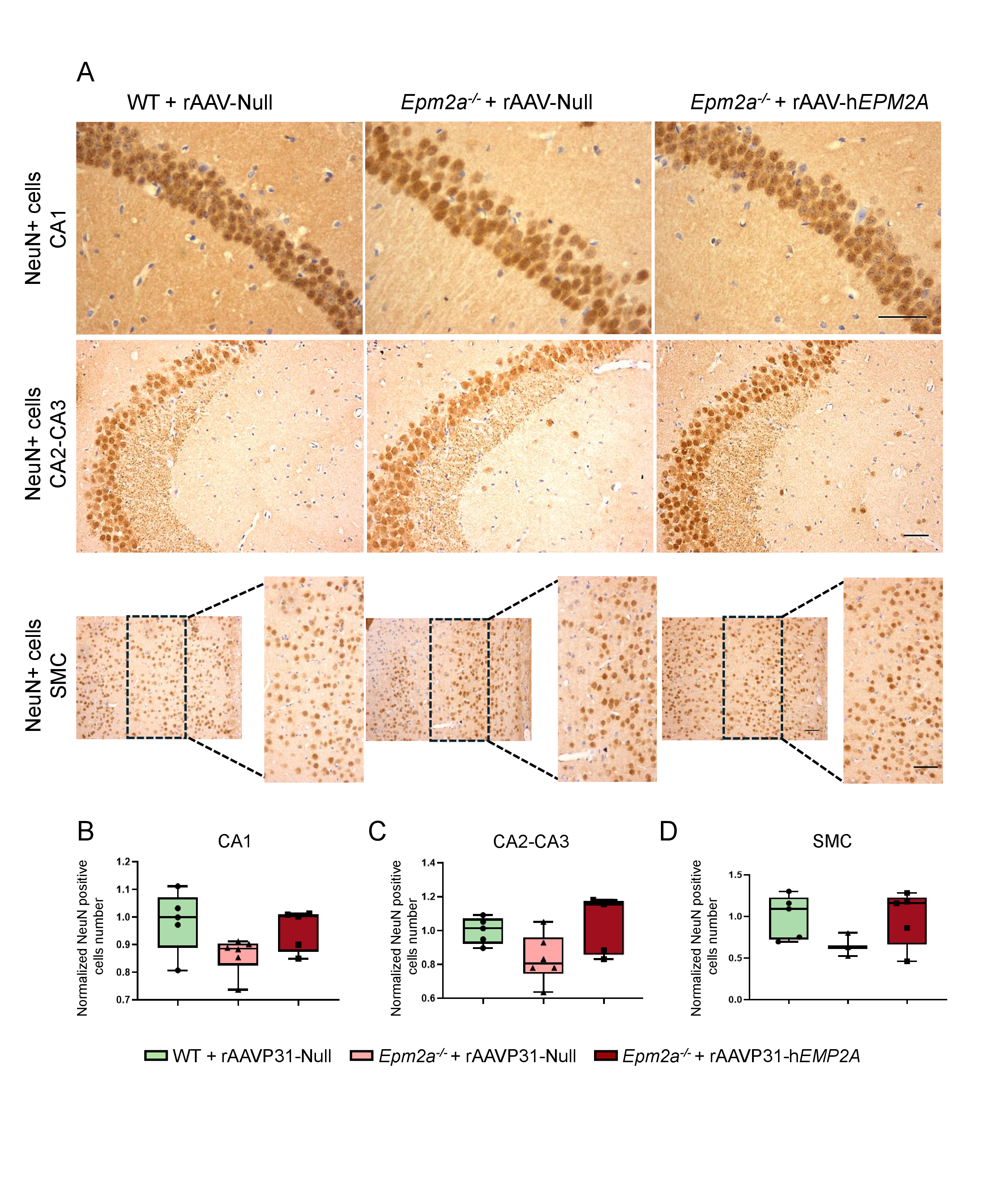


**Supplemental Figure 3. Quantification of NeuN-positive cells in the CA1 and CA2-CA3 region of the hippocampus, and in the SMC of *Epm2a^-/-^* mice IV injected with rAAVP31-h*EPM2A*.** (A) IHC with NeuN antibody for the study of neuronal loss. (B-D) Quantification of neurons in (B) CA1 (WT+rAAVP31-Null vs *Epm2a^-/-^*+rAAVP31-Null *p*-Value=0.1980; WT+rAAVP31-Null vs *Epm2a^-/-^*+rAAVP31-h*EPM2A* *p*-Value=>0.9999; *Epm2a^-/-^*+rAAVP31-Null vs *Epm2a^-/-^*+rAAVP31-h*EPM2A* *p*-Value=0.4074), (C) CA2 (WT+rAAVP31-Null vs *Epm2a^-/-^*+rAAVP31-Null *p*-Value=0.2287; WT+rAAVP31-Null vs *Epm2a^-/-^*+rAAVP31-h*EPM2A* *p*-Value=>0.9999; *Epm2a^-/-^*+rAAVP31-Null vs *Epm2a^-/-^*+rAAVP31-h*EPM2A* *p*-Value=0.1019) and (D) SMC (WT+rAAVP31-Null vs *Epm2a^-/-^*+rAAVP31-Null *p*-Value=0.4083; WT+rAAVP31-Null vs *Epm2a^-/-^*+rAAVP31-h*EPM2A* *p*-Value=>0.9999; *Epm2a^-/-^*+rAAVP31-Null vs *Epm2a^-/-^*+rAAVP31-h*EPM2A* *p*-Value=0.3556) of WT and *Epm2a^-/-^* mice IV injected with rAAVP31-Null or rAAVP31-h*EPM2A* at 6 months of age, 5 months after IV injection. In the SMC, the quantitative analysis was carried out in the enlarged region (width: 747 px; height: 1550 px), corresponding to layers IV-V of the sensorimotor cortex. The results are expressed as the median of independent samples. The bars in the boxplots show the minimum and maximum values. The values were normalized using values from WT mice injected with rAAVP31-Null. A non-parametric Kruskal-Wallis test was performed followed by Dunn's multiple comparisons. * p < 0.05. n = 3-5 mice per group. Scale bar = 50 µm.

**
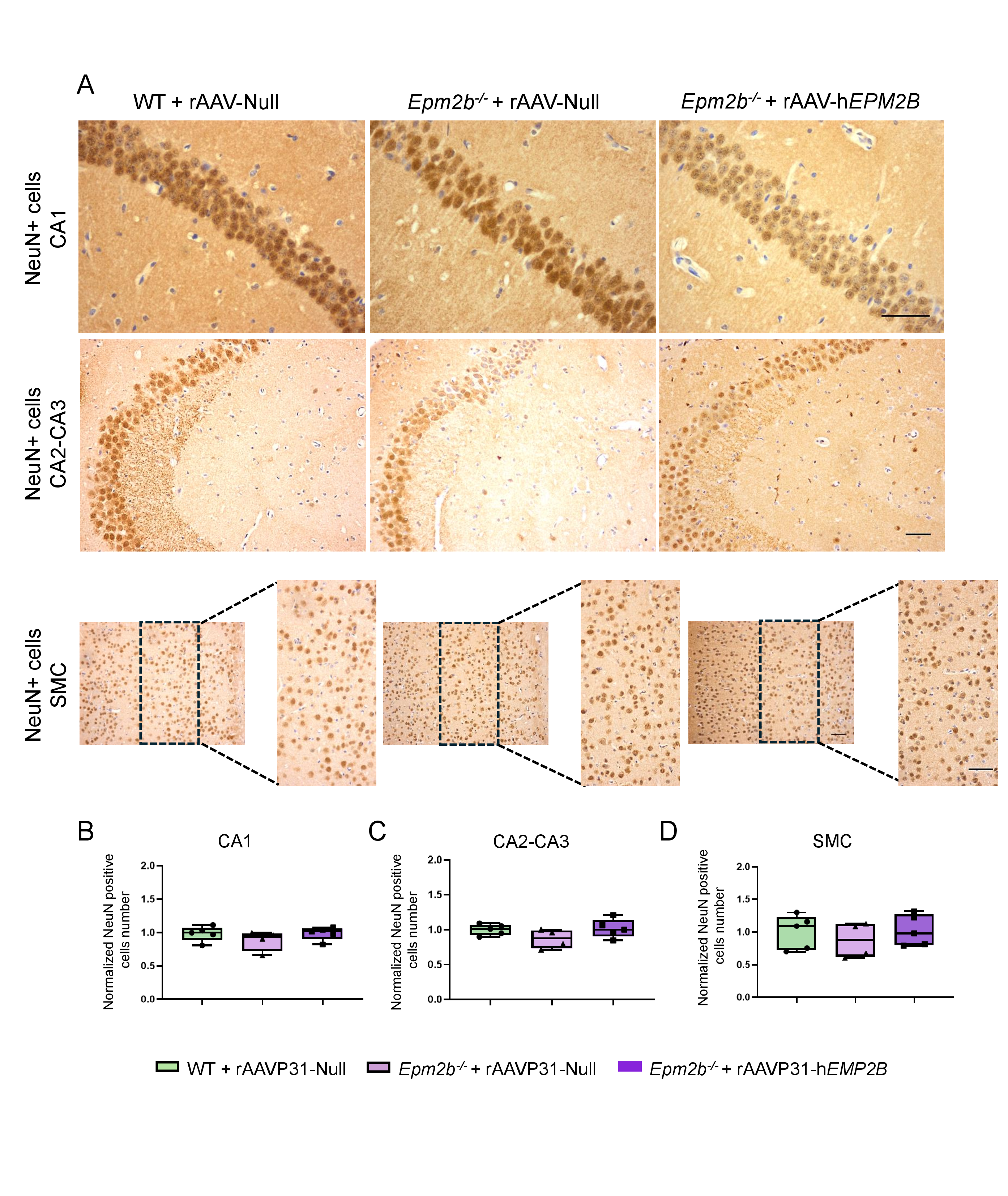
**

**Supplemental Figure 4. Quantification of NeuN-positive cells in CA1 and CA2-CA3 regions of the hippocampus, and in layers IV-V of SMC of IV treated *Epm2b^-/-^* mice.** (A) IHC with NeuN antibody for studying neuronal loss. (B-D) Quantification of neurons in (B) CA1 (WT+rAAVP31-Null vs *Epm2b^-/-^*+rAAVP31-Null *p*-Value=0.4938; WT+rAAVP31-Null vs *Epm2b^-/-^*+rAAVP31-h*EPM2B* *p*-Value=>0.9999; *Epm2b^-/-^*+rAAVP31-Null vs *Epm2b^-/-^*+rAAVP31-h*EPM2B* *p*-Value=0.3264), (C) CA2 (WT+rAAVP31-Null vs *Epm2b^-/-^*+rAAVP31-Null *p*-Value=0.4294; WT+rAAVP31-Null vs *Epm2b^-/-^*+rAAVP31-h*EPM2B* *p*-Value=>0.9999; *Epm2b^-/-^*+rAAVP31-Null vs *Epm2b^-/-^*+rAAVP31-h*EPM2B* *p*-Value=0.3739), and (D) layers IV-V of the SMC (WT+rAAVP31-Null vs *Epm2b^-/-^*+rAAVP31-Null *p*-Value=0.9813; WT+rAAVP31-Null vs *Epm2b^-/-^*+rAAVP31-h*EPM2B* *p*-Value=>0.9999; *Epm2b^-/-^*+rAAVP31-Null vs *Epm2b^-/-^*+rAAVP31-h*EPM2B* *p*-Value=0.6176) of WT and *Epm2b^-/-^* mice IV injected with rAAVP31-Null or rAAVP31-h*EPM2B* at 6 months of age, 5 months after IV injection. The quantitative analysis in the SMC was carried out in the enlarged region (width: 747 px; height: 1550 px), corresponding to layers IV-V of the sensorimotor cortex. Results are expressed as the median of independent samples. The bars in the boxplots show the minimum and maximum values. The values were normalized using values from WT mice injected with rAAVP31-Null. A non-parametric Kruskal-Wallis test was performed followed by Dunn's multiple comparisons. * p < 0.05. n = 4-6 mice per group. Scale bar = 50 µm.

**
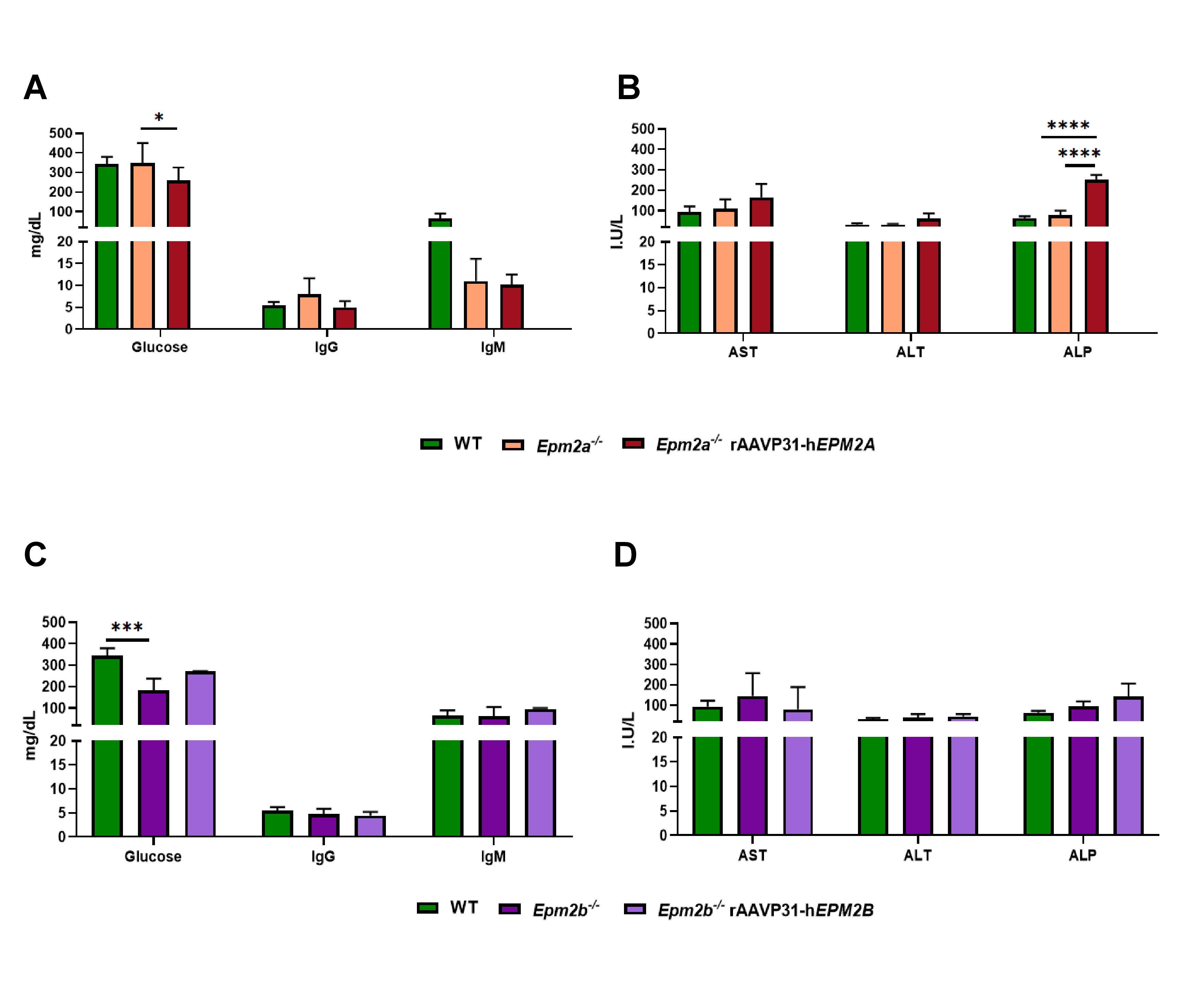
**

**Supplemental Figure 5. Serum liver and immune biochemical parameters in WT, *Epm2a^-/-^* and *Epm2b^-/-^* mice 16-19 months after IV injection.** (A-B) Long-term effects of rAAVP31-*EPM2A* and rAAVP31-h*EPM2B* IV administration on hepatic function and adaptative immune response. Data are shown as mean ± SEM. Statistical analysis was conducted using a two-way ANOVA with Tukey's multiple comparisons test. * p < 0.05. n= 2-4 mice per group.
